# Electro-acoustic coupling of sequential pulsed electric field-ultrasound treatment: A novel technique for modification of protein isolate extracted from *Capsicum annum* L. seeds^☆^

**DOI:** 10.1016/j.ultsonch.2026.108060

**Published:** 2026-09-13

**Authors:** Muhammad Faisal Manzoor, Xin-An Zeng, Zahoor Ahmed, Rana Muhammad Aadil, Silvia del Carmen Beristain-Bauza, Abderrahmane Aït-Kaddour, Muhammad Talha Afraz, Marwa Ezz El-Din Ibrahim, Muhammad Waseem

**Affiliations:** a Guangdong Provincial Key Laboratory of Intelligent Food Manufacturing, School of Food Science and Engineering, Foshan University, Foshan 528225, China; b School of Food Science and Engineering, South China University of Technology, Guangzhou 510641, China; c Human Nutrition and Dietetics, School of Food and Agricultural Sciences, University of Management Technology Lahore, Pakistan; d National Institute of Food Science and Technology, University of Agriculture, Faisalabad, Pakistan; e Energy Engineering, Polytechnic University of Amozoc, Amozoc de Mota, Puebla, Mexico; f Université Clermont Auvergne, INRAE, VetAgro Sup, UMRF, 63370, France; g Deptartment of Food Science and Technology, IPB University, IPB Dramaga Campus, Bogor 16680, West Java, Indonesia; h Department of Food and Nutrition Sciences, College of Agricultural and Food Sciences, King Faisal University, Al-Ahsa 31982, Saudi Arabia; i Department of Food Science & Technology, The Islamia University of Bahawalpur, Punjab, Pakistan

**Keywords:** Pepper seeds protein isolate, Sequential ultrasound-pulsed electric field, Structural modification, Aggregate disaggregation, Techno-functional properties, *In vitro* digestibility

## Abstract

The agro-industrial valorization of pepper (*Capsicum annum* L*.*) seeds as a sustainable protein resource requires innovative processing strategies to overcome the limitations of their native protein structure and enhance their techno-functional properties for food applications. The study aims to investigate the impact of electro-acoustic coupling between sequential pulsed electric field (PEF) and ultrasound (US) treatment on the *in vitro* digestibility, structural, functional, and antioxidant properties of underexploited pepper seed protein isolate (PSPI). The sequential treatment caused significant conformational and structural changes, as shown by increased surface hydrophobicity and free-SH content, decreased particle size (604.82 ± 7 to 275.97 ± 4 nm), and an increase in the magnitude of zeta potential (negative), indicating improved dispersion stability. Intrinsic fluorescence presented a decrease in intensity and a distinct red shift, suggesting exposure of aromatic residues and conformational unfolding. FTIR analysis verified the rearrangements of secondary structure with an increase in random coil and α-helix contents. The scanning electron microscope confirmed these findings, showing significant fragmentation and disruption in SEM images. XRD analysis indicated a reduction in peak intensity (2θ) from 20.12° to 19.71°, showing decreased crystallinity and molecular order. Thermal properties analysis by differential scanning calorimetry revealed a decrease in the enthalpy change (126.75 ± 0.65 to 100.27 ± 0.84 ΔH J/g), indicating reduced thermal stability due to structural modifications. These structural and molecular modifications significantly increased the solubility (48.4 ± 0.59 to 75.2 ± 0.65 %), oil-holding capacity (5.68 ± 0.06 to 7.30 ± 0.07 g/g), water-holding capacity (2.69 ± 0.04 to 3.82 ± 0.05 g/g), emulsification, and foaming properties, while reducing turbidity. Moreover, sequential treatment also significantly improved *in vitro* protein digestibility and antioxidant activity compared with individual PEF and US treatments. Conclusively, the effectiveness of sequential PEF and US as a novel green and sustainable industrial tool to effectively modify PSPI structure to upgrade it into a high-value functional ingredient for food applications.

## Graphical abstract

Graphical representation of electro-acoustic coupling between sequential pulsed electric field-ultrasound treatment for the modification of protein isolate extracted from *Capsicum annum* L. seeds.

**Figure:**
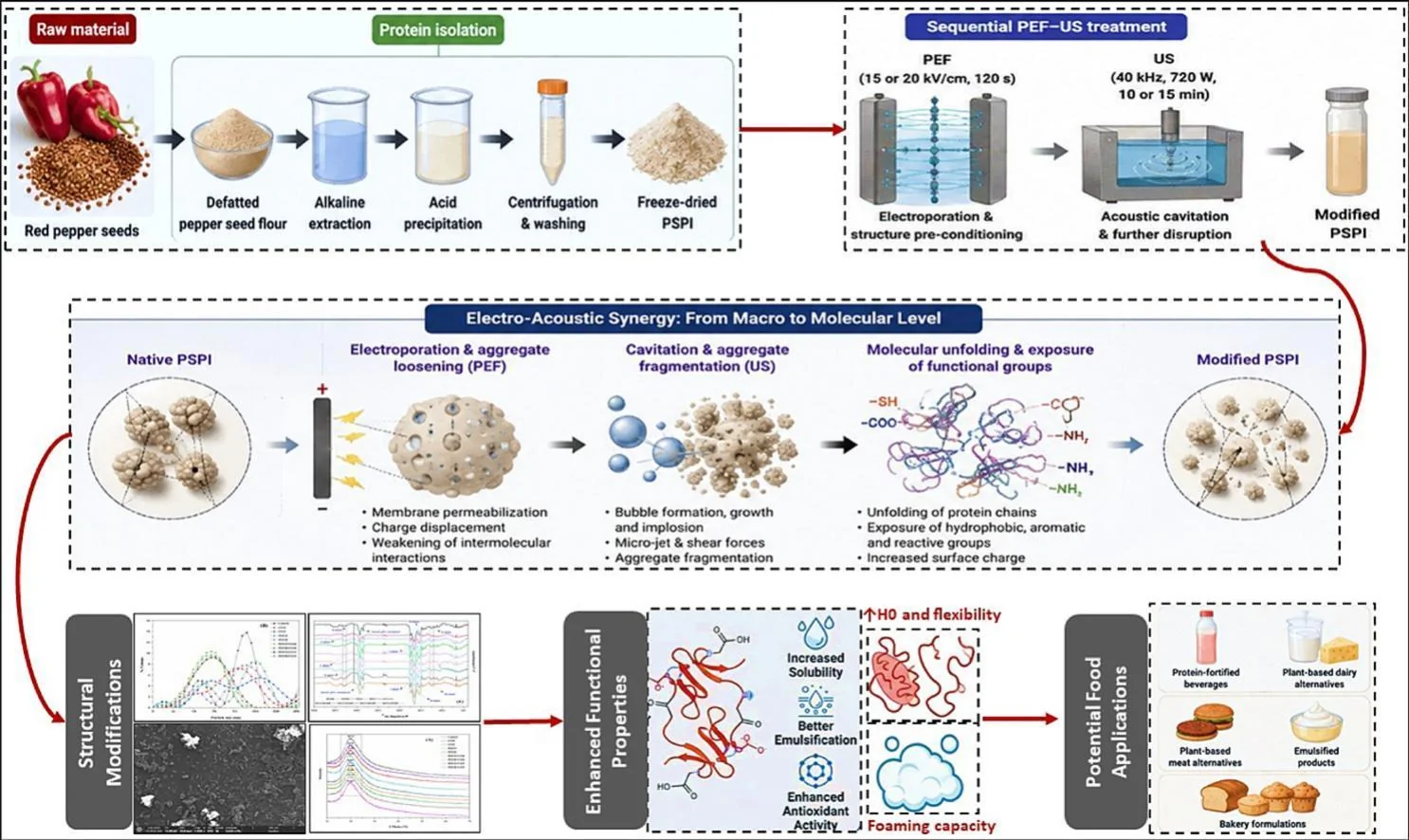

## 1 Introduction

The growing global demand for novel plant dietary proteins and the need to valorize food by-products have shifted researchers' focus to underutilized edible fractions of agro-industrial food waste, such as seed fractions, which are viable carriers of dietary proteins [1]. Pepper (*Capsicum spp*.) seeds are nutrient-rich by-products of the spice industry. It is crucial to valorize pepper seeds and their protein isolates to mitigate the ever-rising challenges of food waste, hidden hunger, and health issues owing to their remarkable capacity to provide healthier proteins with promising techno-functionalities and antioxidant potential [2]. However, the native proteins obtained from seeds exhibit suboptimal foaming/emulsifying properties, as well as poor digestibility and solubility, thereby limiting their viable application in food matrices.

Non-thermal processing approaches suggest tailoring plant protein functionalities by non-destructively improving their structures. In this connection, pulsed electric field (PEF) is a viable approach that delivers microseconds to milliseconds of high-voltage pulses to improve protein conformational rearrangements via transient pore formation in cells, electrodynamic forces, and modulation of protein-solvent interactions [3]. Structural rearrangements and agitation can enhance techno-functional properties such as gelation, foaming, solubility, and surface activity (e.g., water- and oil-holding capacities) and even demonstrate significant improvements in nutritional quality [4], [5]. Recent studies have shown that PEF can regulate bio-macromolecular aggregation states by promoting aggregate disassembly, modifying intermolecular interactions, and inducing conformational rearrangement through non-thermal mechanisms [6], [7]. These findings highlight the potential of PEF for tailoring protein structures and improving functional properties; however, its application for structural regulation of pepper seed protein isolate (PSPI) remains unexplored. Ultrasound (US) is another non-thermal processing technology that offers measurable outcomes in modifying plant proteins by generating acoustic cavitation, microstreaming, and rapidly mobilized shear forces. The US effects consequently disrupt protein aggregates, reveal hydrophobic ends and reactive functional groups, thereby enhancing limited hydrolysis accompanied by partial unfolding of molecular proteins [8], [9]. Moving forward, it is also noteworthy that US treatment improves the oil-holding capacity, emulsifying capability, and antioxidant potential of plant seed protein isolates, resulting from alterations in secondary and tertiary structures and increased surface hydrophilicities [10]. Earlier, Manzoor, Waseem, Diana, Wang, Ahmed, Ahmed, Ali and An-Zeng [9] showed that US treatment can modify red pepper seed protein isolate by inducing cavitation-driven structural changes and improving functional properties at different pH values (7 and 9). However, the effects of US may be enhanced by combining it with other non-thermal technologies; therefore, a sequential PEF+US approach was chosen.

Among the available non-thermal processing technologies, including high-pressure homogenization, cold plasma, and high hydrostatic pressure, US and PEF were selected in this study because they modify protein structures through complementary mechanisms while maintaining mild processing conditions. PEF primarily induces conformational rearrangements and alters intermolecular interactions through electric field-mediated effects [11], whereas US generates cavitation, microstreaming, and shear forces that facilitate protein unfolding, dispersion, and aggregate disruption [12]. Although other non-thermal technologies have also demonstrated effectiveness in modifying plant proteins, a direct comparison among technologies was beyond the scope of the present study. Consequently, the sequential application of PEF and US may produce synergistic effects on protein structure and functionality that cannot be achieved by either treatment alone. The electro-acoustic coupling between sequential PEF+US processing derives from the interaction between electric-field-generated molecular polarization and cavitation-induced mechanical forces, whereby PEF lowers cavitation thresholds. It destabilizes protein conformations, allowing US to generate microstreaming, intensified shear, and localized energy escape, thereby enhancing structural changes and improving the functional properties of plant proteins [12]. Earlier literature on the synergistic use of combined PEF+US has also reported significant improvements in the extraction yields of intracellular compounds, mass transfer, and the recovery and retention of bioactive compounds compared with using these technologies alone [13]. Further, the preliminary scientific exploration across complex food matrices suggests that the simultaneous or sequential use of PEF+US can also enhance the techno-functional and structural elucidation, without revealing any processing losses [14]. However, despite the number of research works, a systematic study demonstrating the synergistic use of PEF+US to investigate structural metrics such as secondary structure, antioxidant activity, and digestibility traits remains missing for plant proteins.

Although the combined application of PEF and US has recently been explored for the modification of certain plant proteins, such as chickpea protein isolate [12], its potential effects on pepper seed protein isolate (PSPI) remain unexplored. PSPI is an underutilized protein source obtained from pepper seed processing by-products and possesses distinct compositional and structural characteristics that may influence its response to non-thermal processing. Therefore, the present study investigates the effects of sequential PEF+US treatment on the structural, physicochemical, thermal, and functional properties of PSPI, providing insights into the valorization of pepper seed by-products as sustainable protein ingredients.

## 2 Materials and Methods

### 2.1 Sample preparation and defatting

Dried pepper (*Capsicum spp*.) seeds were purchased from the local market of Guangzhou, Guangdong, China. The procured seeds were ground into flour using a high-speed crusher (Wenling, China, DFY-500C) to remove coarse particles, and the flour was passed through a 40-mesh sieve. After that, *n*-hexane was added to the flour at a 1:5 w/v ratio and continuously stirred for 24 h. Then, defatted flour was first dried at room temperature, followed by drying in a hot-air oven at 50 °C for 2 h. The defatted dried flour was packed in polyethylene bags and stored at ambient temperature until further processing. The defatted pepper seed flour contained 1.96±0.08 % moisture, 24.87±0.22 % protein, 1.79±0.09 % crude fat, 4.98±0.31% ash, and 66.40±0.23 % carbohydrate.

### 2.2 Preparation of pepper seed protein isolate

The pepper seed protein isolate (PSPI) was prepared according to Deng, Du, Liu, Xiong, Wang, Rao, Liu, Zhao and Liao [15] using alkali and acid precipitation. Defatted flour (250 g) was dispersed in distilled water (5 L, 1:20 w/v), and the pH was adjusted to 11 with 1 M NaOH. The obtained mixture was then gently stirred at room temperature for 7 h. After that, the solution was centrifuged at 9000×g for 15 min at 4 ℃, and the supernatant was filtered. The pH of the supernatant was adjusted to 4.5 (1 M HCl), and the solution was centrifuged again at 9000×g for 15 min at 4 ℃ to obtain precipitates. After that, the precipitates were washed with deionized water and neutralized to pH 7. The precipitates were then dissolved in deionized water to a final volume of 100 mL and dialyzed at 4 ℃ for 24 h against Milli-Q ultrapure H_2_O using a 3500 Da molecular cutoff membrane. The obtained PSPI solution was freeze-dried for 48 h at -80 ℃ and stored at -20 ℃ until further use. The extraction yield of PSPI was 6.08±0.11% (w/w, based on defatted flour DW), with a protein purity of 82.02±0.21%.

The PSPI concentration was adjusted to 8 mg/mL based on previous studies and analytical requirements to ensure stable protein dispersion and reliable structural and functional characterization. This concentration was maintained throughout the study to consistently evaluate the effects of PEF, US, and sequential treatments.

### 2.3 PSPI processing

#### 2.3.1 Pulsed Electric Field (PEF)

A 140 mL PSPI solution (8 mg/mL) was prepared in ultrapure water and mixed well before treatment. For PEF treatment, a continuous system, manufactured by Guangzhou Pahu Technology Co., China, was used. The device specifications are as follows: power 2.5 kW, input voltage 0-20 kV, output voltage 220 kV/50 Hz, Y-Z-500, cylindrical chamber, separating metal electrodes with plate electrodes (0.30 cm with 0.02 mL of volume), at a pulsed width of 40 µs with a frequency of 1 kHz. An electric roller pump was used to deliver the 140 mL PSPI solution into the treatment chamber at 180 mL/min. The samples were treated with PEF at 15 kV/cm and 20 kV/cm for 120 seconds; PEF-treated samples were coded as 15 kV/cm (PEF15) and 20 kV/cm (PEF20). The temperature of the treated samples was maintained at 30±2 °C during treatment using a water-circulating condensation system. After that, the PEF-treated samples were lyophilized and stored at ambient temperature for further analysis. The PEF treatment conditions were selected based on preliminary screening experiments and previous studies demonstrating that moderate electric field strengths effectively induce structural modifications in proteins while minimizing excessive aggregation or degradation [11], [16]. During the preliminary screening, electric field strengths of 10, 15, 20, and 25 kV/cm were evaluated. Based on these observations and literature reports, field strengths of 15 and 20 kV/cm applied for 120 s were selected because these electric field strengths are reported to facilitate disruption of non-covalent interactions, partial unfolding, and molecular polarization, thereby improving protein reactivity. The treatment time was optimized to ensure sufficient energy input under non-thermal conditions.

#### 2.3.2 Ultrasound (US)

A 50 mL PSPI solution (8 mg/mL) was prepared in ultrapure water and poured into a flat-bottom flask (100 mL). The samples were then ultrasonicated in an US bath system (JP-070S, Skyman Corporation, China). The sample treatment was performed at 40 kHz, 720W at 30±2 °C for 10 and 15 min, respectively. The nominal power density, calculated as the rated electrical power divided by the actual water volume in the bath, was 120 W/L (0.12 W/mL). Based on this, the nominal power available to the 50 mL sample volume was 6.0 W. All positional parameters, water volume, and temperature settings were kept constant across all experiments to ensure reproducibility. After the US treatment, the samples were coded as US10 and US15. The US-treated samples were then lyophilized and stored at ambient temperature for further analysis.

The US treatment durations were selected based on preliminary screening experiments and previous studies reporting that moderate sonication effectively modifies protein structure through cavitation, while prolonged treatment may lead to excessive aggregation or structural deterioration [9], [12]. Initially, US treatment times of 5, 10, 15, and 20 min were evaluated. Based on these observations and literature findings, treatment durations of 10 and 15 min were selected for further investigation. The 10-min treatment promotes optimal protein dispersion and unfolding, while the 15-minute treatment estimates the impact of prolonged exposure. It permits evaluation of the transition from beneficial alteration to excessive processing, especially following PEF pretreatment.

#### 2.3.3 Sequential treatment

Based on previous studies, the sequential treatment was designed by applying PEF before US because PEF serves as a mild pretreatment that induces partial protein unfolding and weakens intermolecular interactions, thereby facilitating the penetration and cavitation effects of subsequent US. This sequence was selected to investigate the potential synergistic effects of PEF pretreatment on US-induced structural modification and functional improvement of PSPI. The reverse sequence (US→PEF) was not included because comparing treatment order was beyond the scope of the present study; however, its effects may differ and warrant investigation in future studies. For sequential treatment, the PSPI solution (8 mg/mL) was prepared in ultrapure water. First, PEF was performed at 15 kV/cm (PEF15) and 20 kV/cm (PEF20) for 120 seconds, followed by US treatment (10 min (US10) and 15 min (US15)). The sequential treatment samples were coded as PEF15+US10, PEF20+US10, PEF15+US15, and PEF20+US15 (sample codes are further described in the footnotes to Table 1, Table 2). After sequential treatments, all samples were cooled in a water bath and then lyophilized for further analysis.

**Table 1:** Impact of US, PEF, and sequential treatments on particle size, zeta potential, and IVPD (%) of PSPI.

Samples	Particle size (nm)	Zeta potential (mV)	IVPD (%)
Control	604.82 ± 7^a^	−52.38 ± 0.18^a^	82.23 ± 0.58^e^
US10	469.34 ± 6^d^	−55.92 ± 0.21^bc^	84.68 ± 0.63^cd^
US15	455.12 ± 4^de^	−56.43 ± 0.23^cd^	85.32 ± 0.73^c^
PEF15	567.43 ± 5^b^	−54.61 ± 0.17^b^	83.12 ± 0.78^d^
PEF20	525.51 ± 2^c^	−55.82 ± 0.19^bc^	83.96 ± 0.45^cd^
PEF15 + US10	333.53 ± 6^e^	−56.96 ± 0.26^cd^	85.82 ± 0.52^bc^
PEF20 + US10	315.24 ± 8^ef^	−57.25 ± 0.16^d^	86.12 ± 0.48^b^
PEF15 + US15	275.97 ± 4^fg^	−58.32 ± 0.24^e^	89.02 ± 0.38^a^
PEF20 + US15	285.68 ± 3^f^	−57.82 ± 0.22^d^	85.12 ± 0.43^c^

All the results (n = 3) are presented as mean ± S.D., while the different alphabets (a-g) among columns show the significant difference (*p* < 0.05) between treatments and control (Tukey’s HSD).

**Control:** Untreated sample, **US10:** Ultrasound treatment for 10 mins, **US15:** Ultrasound treatment for 15 mins, **PEF15:** Pulsed electric field treatment at 15 kV for 2 mins, **PEF20:** Pulsed electric field treatment at 20 kV for 2 mins, **PEF15 + US10:** Pulsed electric field treatment at 15 kV then ultrasound for 10 mins, **PEF20 + US10:** Pulsed electric field treatment at 20 kV then ultrasound for 10 mins, **PEF15 + US15:** Pulsed electric field treatment at 15 kV then ultrasound for 15 mins, and **PEF20 + US15:** Pulsed electric field treatment at 20 kV then ultrasound for 15 mins.

**Table 2:** Impact of US, PEF, and sequential treatments on thermal properties of PSPI.

Samples	Onset T_o_ (℃)	Denaturation T*_d_* (℃)	Offset T*_e_* (℃)	Enthalpy ΔH (J/g)
Control	46.29 ± 0.57^d^	89.00 ± 0.32^a^	143.83 ± 0.54^b^	126.75 ± 0.65^a^
US10	46.80 ± 0.66^d^	85.82 ± 0.58^d^	144.53 ± 0.62^a^	108.88 ± 0.56^d^
US15	49.75 ± 0.48^a^	89.30 ± 0.87^a^	144.04 ± 0.35^a^	107.13 ± 0.59^d^
PEF15	48.47 ± 0.45^b^	85.16 ± 0.71^d^	144.26 ± 0.43^a^	123.79 ± 0.78^b^
PEF20	49.58 ± 0.53^a^	87.98 ± 0.68^b^	144.16 ± 0.62^a^	121.69 ± 0.54^c^
PEF15 + US10	47.32 ± 0.72^c^	86.94 ± 0.92^c^	144.22 ± 0.87^a^	105.88 ± 0.48^e^
PEF20 + US10	48.98 ± 0.33^b^	86.94 ± 0.54^c^	144.13 ± 0.78^a^	103.76 ± 0.66^f^
PEF15 + US15	47.75 ± 0.43^c^	81.99 ± 0.67^e^	141.19 ± 0.91^a^	100.27 ± 0.84^g^
PEF20 + US15	47.57 ± 0.39^c^	80.42 ± 0.45^f^	144.40 ± 0.93^a^	103.34 ± 0.34^f^

All the results (n = 3) are presented as mean ± S.D., while the different alphabets (a-g) among columns show the significant difference (*p* < 0.05) between treatments and control (Tukey’s HSD).

**Control:** Untreated sample, **US10:** Ultrasound treatment for 10 mins, **US15:** Ultrasound treatment for 15 mins, **PEF15:** Pulsed electric field treatment at 15 kV for 2 mins, **PEF20:** Pulsed electric field treatment at 20 kV for 2 mins, **PEF15 + US10:** Pulsed electric field treatment at 15 kV then ultrasound for 10 mins, **PEF20 + US10:** Pulsed electric field treatment at 20 kV then ultrasound for 10 mins, **PEF15 + US15:** Pulsed electric field treatment at 15 kV then ultrasound for 15 mins, and **PEF20 + US15:** Pulsed electric field treatment at 20 kV then ultrasound for 15 mins.

### 2.4 Structural characterization

#### 2.4.1 Surface hydrophobicity (H_0_)

The US, PEF, and sequentially treated and control PSPI samples were tested for H_0_ using the procedure as outlined by Yang, Liu, Ren, Huang, Huang and Zhang [17]. The PSPI sample was mixed with distilled water at 0.01-0.5 mg/mL. Thereafter, 8 mmol/L of the 8-anilinonaphthalene-1-sulfonic acid (ANS) solution (20 μL) was mixed with an accurate volume of 4 mL of PSPI solution. The fluorescence spectrophotometer (Onlab EU-2600 A, China) was used to measure the fluorescence intensities of each sample and the reagent blank at 463 and 360 nm for emission and excitation states, respectively. Subsequently, the fluorescence intensities at the initial slope were plotted against protein concentration to estimate a surface hydrophobicity index.

#### 2.4.2 Free sulfhydryl group (-SH)

The method of Zhao, Liu, Zhao, Ren and Yang [18] was followed to estimate free-SH groups in the US, PEF, and in sequentially-treated and control PSPI samples. Specifically, about 1 mL of the triglycine buffer holding 7.0 pH (i.e., 0.09 M glycine, 0.086 M Tris, and 0.004 mM EDTA) was amalgamated with accurately measured 4 mg of 5, 5′- dithiobis-2-nitrobenzoic acid (DTNB) for the preparation of Ellman’s solution. Thereafter, the lyophilized PSPI solutions were amalgamated in a 0.15% (*w/v*) Tris-glycine buffer solution and 50-μL of Ellman’s reagent, which was already mixed in the 5 mL protein suspension. Consequently, the protein mixture was placed in a water bath shaker for incubation at 25±1 °C for 1 h. The admixtures were centrifuged at 10,000 x *g* for 10 min at room temperature. Spectrophotometric (Onlab EU-2600 A, China) absorbance of the sample, reagent blank (i.e., buffer solution), and standard was recorded at 412 nm, using a molar extinction coefficient of 13,600 M/cm to measure the free sulfhydryl content (i.e., μmol/g protein).

#### 2.4.3 Zeta potential and average particle size

Zeta potential and particle size of US, PEF, and sequentially treated and control PSPI samples were measured as described by Lu, Xiong, Yao, Zhang and Wang [19]. The PSPI solution was prepared at 10 mg/mL before centrifugation at 12,000 *g* for 15 min at 10 °C. Subsequently, about 2 mL of each supernatant was diluted to 0.1 mg/mL and determined for zeta potential and average particle size using the Zetasizer Nano (Malvern Panalytical, UK) at a temperature of 25 °C and a refractive index (γ) of 1.330.

#### 2.4.4 Intrinsic fluorescence (IF)

The IF of the US, PEF, and sequentially treated samples was estimated using the standard protocol outlined by He, Chen, He, Feng, Li, Liu, Dai and Liu [20]. Each PSPI sample solution was diluted to 0.15 mg/mL in phosphate buffer saline (PBS). Thereafter, the PSPI sample fluorescence spectra were individually obtained using a fluorescence spectrophotometer (Hitachi F-4600, Kyoto, Japan) at an excitation wavelength of 280 nm, slit widths of 5 nm, an emission range of 300-500 nm, and a scanning speed of 1000 nm/min. Each experiment was done in triplicate.

#### 2.4.5 Fourier Transform Infrared (FTIR) Spectroscopy

To observe changes in the secondary structures of US, PEF, and sequentially treated PSPI samples, FTIR spectroscopy was used as described by Sui, Sun, Qi, Zhang, Li and Jiang [21] with slight modifications. Precisely measured 2 mg of each PSPI vacuum freeze-dried (Alpha 1-2 D Plus, Marin Christ, Germany) sample was mixed with 200 mg of potassium bromide (KBr) and thoroughly ground in an agate mortar to obtain a transparent flake sample. Thereafter, the FTIR spectra were recorded on an FTIR spectrophotometer (Perkin Elmer, UK), over 400-4000 cm^-1^, with a resolution of 4 cm^-1^. Then, the amide-I bands in each spectrum (1600-1700 cm^-1^) were measured by PeakFit software, and a Gaussian function on Origin 8.0 was used to measure the secondary structure fraction of each sample.

#### 2.4.6 Scanning Electron Microscopy (SEM)

The lyophilized PSPI samples were initially coated with a 15 nm-thick gold layer, using an ion sputter (Hitachi E-1010, Japan). Thereafter, all the samples were analyzed using a field-emission scanning electron microscope (Thermo Fisher Scientific, Quattro S, Brno S.R.O., Vlastimila Pecha, Czech Republic) at an accelerating voltage of 5 kV and a magnification of 1000X.

#### 2.4.7 X-Ray Diffraction (XRD)

For XRD analysis, a Bruker D8 DISCOVER diffractometer was used, coupled with a rotating copper anode X-ray tube functioning at 80 mA and 40 kV. The diffractometer employs Cu Kα (λ=1.5418 Å) radiation, and the graphite monochromator produces high-intensity diffraction patterns over the 2θ and 10-70° ranges. This is done at a step size of 0.03° and a dwell time of 1 s per step. Subsequently, phase identification was performed using the X-ray qualitative analysis software, i.e., JADE (Materials Data Inc., Livermore, CA, USA).

### 2.5 Differential Scanning Calorimetry (DSC)

All the PSPI samples were freeze-dried and analyzed by DSC (DSC-8000, PerkinElmer, USA). Each PSPI sample was heated from 30 to 150 °C at 10 °C/min under nitrogen carrier gas.

### 2.6 Functional Characterization

#### 2.6.1 Solubility

The solubility of the US, PEF, and sequentially treated PSPI samples was measured using the Biuret method as documented by Gulzar, Martín-Belloso and Soliva-Fortuny [22]. Specifically, 10 mg/mL of each PSPI sample was dispersed in distilled water and stirred for 1 h with a magnetic stirrer (at 1000 rpm). The samples were then centrifuged for 20 min at 10,000 g at ambient temperature. One portion of each sample supernatant was then mixed with five portions of Biuret solution and incubated at ambient temperature for 30 min. Absorbances of each sample, reagent blank, and standard (bovine serum albumin) were recorded at 540 nm for determining the protein concentrations in the PSPI samples. Thereafter, the protein solubility of each PSPI sample was documented as % protein remaining in the solution.

#### 2.6.2 Turbidity

The turbidity of the US, PEF, and sequentially treated RSPI and control samples was determined following the method of Malik, Sharma and Saini [23], by preparing 1% protein dispersions in distilled water. Subsequently, the turbidity of each sample was recorded using a UV-Vis spectrophotometer at 600 nm at room temperature.

#### 2.6.3 Oil holding capacity (OHC) and water holding capacity (WHC)

For WHC and OHC of all PSPI samples, briefly, 1 g of each PSPI sample was accurately weighed and thoroughly mixed with 40 mL of rapeseed oil and distilled water. The admixture was stirred for 1 min, then rested at 20-23 °C for 6 h. Subsequently, all samples were centrifuged at 20 °C for 30 min at 3000 *g*. WHC and OHC were then calculated using the following equation;

(1) $$\mathrm{WHC}/OHC=\frac{W_{0}-W_{1}}{W_{3}}$$

Where, *W*_0_ = Mass of falcon tube & PSPI, and absorbed water/the oil, *W*_1_ = Mass of falcon tube & PSPI, and *W*_3_ = Mass of PSPI

#### 2.6.4 Foaming properties

The PSPI samples treated with US, PEF, and sequential treatments were tested for the foaming capacity (FC) and foaming stability (FS) in accordance with the experimental protocol as documented earlier by Loushigam and Shanmugam [24]. In this, a 1% PSPI sample solution (i.e., pH = 7) was dissolved in distilled water. Subsequently, the reaction mixture was stirred for 1 h, and an accurate volume of 15 mL of the protein mixture was homogenized for 3 min using a homogenizer (IKA T18 Ultra-Turrax Basic, United Kingdom). Afterward, the total volume was measured before and after whipping. The foam layer volume at 15 and 30 min was determined by comparing the initial foam volume of each sample, which was used to estimate FC and FS.

(2) $$FC\left(\%\right)=\frac{\mathrm{Foamvolume}}{15\;mL}\times 100$$

(3) $$FS\left(\%\right)=\frac{Foamvolumeafter15and30min}{\mathrm{Initialfoamvolume}}\times 100$$

#### 2.6.5 Emulsifying activity index (EAI) and emulsion stability index (ESI)

ESI and the EAI of each PSPI treated with US, PEF, and sequential treatment were estimated by following the method of Pearce and Kinsella [25]. In this, about 15 mL of each sample of 0.1% (*w/v*) protein mix was mixed with 5 mL sunflower oil for 10 min at 10000 rpm using a homogenizer (T-25 homogenizer, IKA, Germany) to formulate an emulsion. Thereafter, a precisely measured 50 µL aliquot of the prepared emulsion (0 and 30 min) was diluted to 5 mL (i.e., 100 times) using 0.1% sodium dodecyl sulfate (SDS) solution, and absorbance was taken at 500 nm (Beckman DU 500, USA). All experiments were tested in triplicate (n = 3), and ESI and EAI were measured by following the equations given below;

(4) $$\mathrm{EAI}\left(\frac{m^{2}}{g}\right)=\frac{2\times 2.303\times DF\times A_{0}}{(1-\theta )\times C\times \emptyset \times 10000}$$

(5) $$\mathrm{ESI}\left(\%\right)=\frac{A_{0}}{A_{0}-A_{30}}\times 10$$

Where, A_0_ = Absorbance recorded at 0^th^ min of thinned suspension, DF = Thinning factor (×100), c = Sample level (g/mL), ϕ = Visual path, θ = Portion of oil (0.25), A_30_ = Absorbance post 30^th^ min

### 2.7 Antioxidant characterization

#### 2.7.1 ABTS radical scavenging assay

The ABTS scavenging activity of US, PEF, and sequential-treatment-treated PSPI samples was measured using the method of Kim and Lee [26]. Briefly, about 100 μL of each PSPI sample was thoroughly mixed with 2 mL of ABTS solution and incubated in the dark for 6 min. Microplate reader absorbance was measured at 734 nm, and ABTS scavenging activities were calculated using the following equation:

(6) $$ABTS\left(\%\right)=\frac{A_{\mathrm{blank}}-A_{\mathrm{sample}}}{A_{\mathrm{blank}}}\times 100$$

Where, A*_blank_* = Distilled water mixed in the ABTS reagent, and A*_sample_* = Sample mixed in the ABTS reagent.

#### 2.7.2 DPPH radical scavenging assay

Each PSPI sample was measured for DPPH radical scavenging activity by the approach previously validated by Santos, Brizola and Granato [27]. Therein, 100 μL of DPPH solution (i.e., prepared in 0.2 mM ethanol) was added to the 100 μL PSPI sample, and the mixture was incubated in the dark for 0.5 h. After incubation, each sample was measured for the DPPH absorbance at 517 nm using a Microplate Reader (SpectraMax® i3x, CN). The results for the DPPH % scavenging activities are calculated by;

(7) $$DPPH\left(\%\right)=\frac{A_{\mathrm{blank}}-A_{\mathrm{sample}}}{A_{\mathrm{blank}}}\times 100$$

Where, A*_blank_* = Ethanol solvent mixed in the DPPH reagent, and A*_sample_* = Sample mixed in the DPPH reagent.

#### 2.7.3 Hydroxyl radical scavenging assay

The OH radical-scavenging activities of all PSPI samples (US, PEF, sequential and control) were measured using the methodology previously validated by Zou, Yang, Li, Zhang, Zhang, Xu and Wang [28]. Therein, 1 mL of each ferrous sulfate (FeSO_4_) and 1,10-phenanthroline (0.75 mM) were stirred together in 1 mL of 0.2 M sodium phosphate buffer (pH = 7.4). Thereafter, 1 mL of each PSPI sample (2 mg/mL) and 0.01% hydrogen peroxide (H_2_O_2_) solution were mixed thoroughly. After proper mixing, the admixture was incubated at 37 °C for 60 min, and the absorbance was measured at 510 nm. OH radical scavenging activities of all PSPI samples were determined using the following equation:

(8) $$OHradicalscavenging\left(\%\right)=\frac{A_{1}-A_{2}}{A_{1}-A_{0}}\times 100$$

Where, A_0_ = Absorbance of distilled water instead of the sample (blank group), A_1_ = Absorbance of distilled water instead of the sample & H_2_O_2_ (control group), and A_2_ = Absorbance of the sample group.

### 2.8 *In vitro* protein digestibility (IVPD)

The PSPI solution (8 mg/mL) was adjusted to pH 8.0 with 0.1 M NaOH. A multienzyme solution (pH 8) containing trypsin (1.6 mg/mL), chymotrypsin (3.1 mg/mL), and protease (1.3 mg/mL) was prepared, and casein was used as a control. The prepared multienzyme solution (1.5 mL) was added and properly mixed. After that, the prepared solution was incubated in a water bath at 37 °C for 10 min, and the pH decline was estimated [29]. IVPD was calculated using the empirical equation as follows:

(9) $$IVPD\left(\%\right)=210.46-18.10X_{f}$$

Where: X*_f_* indicates the pH value of the solution (after 10 min). Although this equation is not specifically calibrated for PSPI, it was consistently applied to all samples under identical conditions to evaluate relative treatment effects.

### 2.9 Statistical analysis

All US, PEF, and sequential treatment PSPI samples were analyzed in triplicate (*n*=3), and the results were presented as Mean±S.D. Results were statistically analyzed using one-way Analysis of Variance (ANOVA) with Minitab 14.1 (Minneapolis, USA). When *p*≤0.05 was detected, Tukey’s HSD test was applied to analyze pairwise comparisons between the control, US, PEF, and sequential treatment and among the treatments themselves. Data were plotted using OriginPro (8.0, Minneapolis, USA).

## 3 Results and Discussion

### 3.1 Free -SH contents and Surface hydrophobicity (H_0_)

**Fig. 1:**
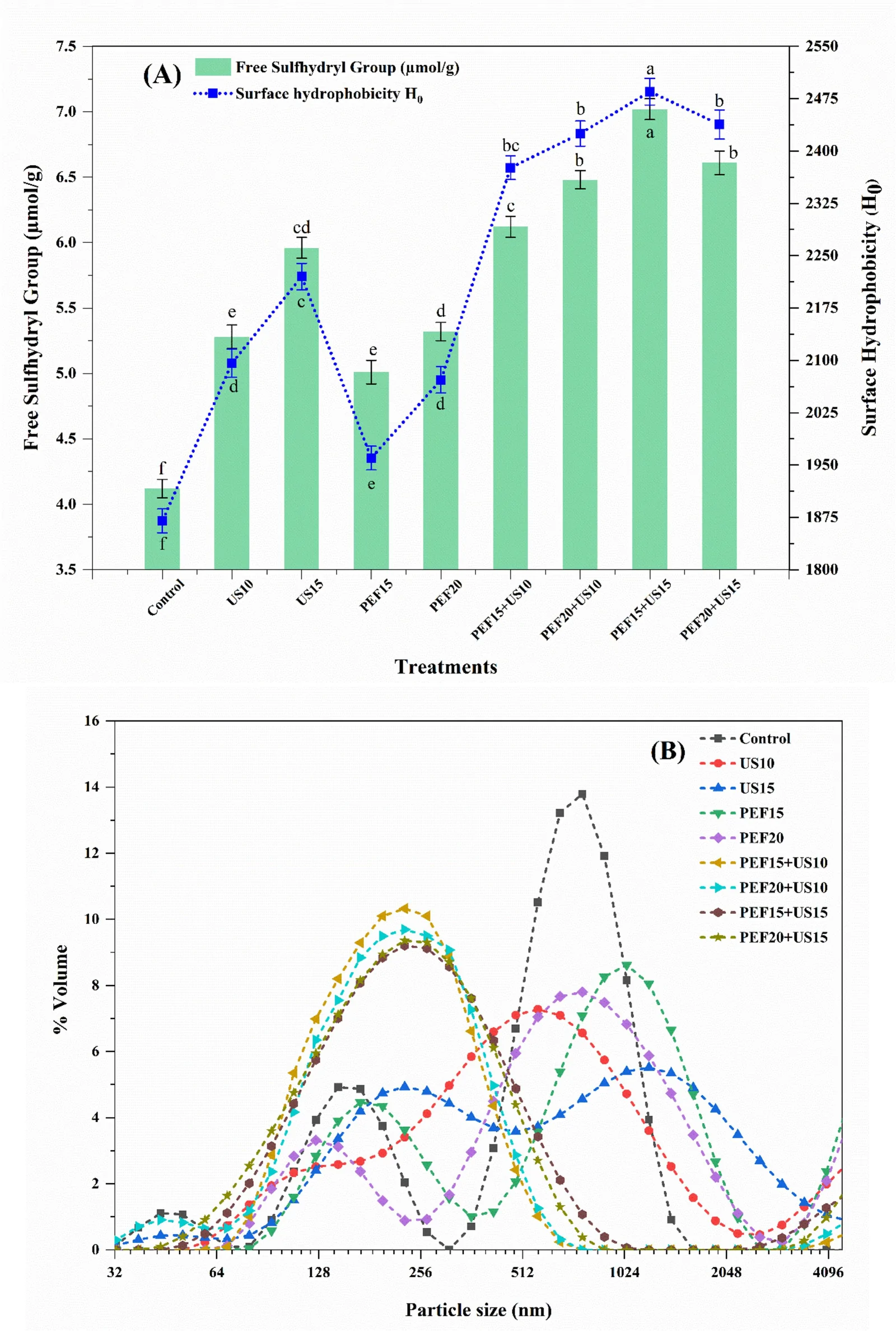
Free sulfhydryl content and surface hydrophobicity **(A)**, and Particle size distribution **(B)** of PSPI after the impact of US, PEF, and sequential treatments. All the results (n = 3) are presented as mean ± S.D., while the different alphabets (a-g) above the bars show the significant difference (*p* < 0.05) between treatments and control (Tukey’s HSD).

Free -SH contents and H_0_ are important parameters that have provided valuable insights into the effect of novel processing techniques on protein conformational modifications. In the current study, US, PEF, and their sequential (PEF+US) treatments significantly (*p<0.05*) enhanced the free-SH contents and H_0_ of PSPI compared with the control sample (4.12±0.07 µmol/g and 1870±17, respectively). Results revealed that individual US treatment induced significant changes in free-SH and H_0_ (5.96±0.08 µmol/g and 2220±19), compared with PEF (5.96±0.08 µmol/g and 2072±18), while the sequential treatment (PEF15+US15) showed the most significant changes (7.02±0.06 µmol/g and 2485±20) (Fig. 1A).

After PEF treatments, an increase in H_0_ is observed due to exposure of previously buried hydrophobic amino acid residues and partial unfolding of protein molecules. PEF disrupts electrostatic interactions and H-bonding without causing excessive protein thermal denaturation, thereby resulting in a moderate conformational alteration [11]. Similarly, a mild improvement in free-SH content after PEF treatment is due to molecular polarization induced by PEF, which exposes -SH groups previously entangled in disulfide linkages by loosening the tertiary structures [22]. Significantly, the comparatively lower free-SH improvement observed with the US treatment reveals that PEF has a minimal impact on covalent bond disruption. Earlier, increases in free-SH and H_0_ of soy protein [11] and faba bean protein isolate [22] were reported after PEF treatment, indicating improved molecular flexibility.

The US treatment significantly increases H_0_ compared with PEF alone, due to induced acoustic cavitation, which produces localized high-pressure, high-temperature zones, microjets, and intense shear forces, leading to aggregate dissociation and protein unfolding. Consequently, the H_0_ groups were more exposed to the aqueous solution [30]. The increased hydrophobic interaction activity after US treatment may be attributed to US-induced cavitation effects, which promote protein unfolding, disrupt intermolecular interactions, and expose previously buried hydrophobic groups. Similar mechanisms have been reported, where US treatment facilitated conformational rearrangement and increased the accessibility of hydrophobic regions in proteins by Qian, Chen, Zhang, Gao, Xu, Guan and Wang [31]. US processing induced a more significant increase in free-SH content, associated with transient radical formation and mechanical stress from cavitation, thereby facilitating protein chain unfolding and disulfide bond disruption [22]. The exposure of free-SH content shows improved PSPI structural reactivity and flexibility, which is ultimately linked with enhanced functional properties. Similar results have been reported for pea protein [30] and faba bean protein isolates [22] after US treatment for free-SH content and H_0_.

Notably, the sequential treatments, specifically PEF15+US15, yielded the highest free-SH content and H_0_, suggesting an apparent synergistic effect. Firstly, PEF pretreatment might partially unfold protein molecules and weaken protein-protein bindings, making them more open to later US-induced cavitation. This successive treatment increases disruption of the disulfide bond and significant exposure of the hydrophobic group compared with individual treatments (US and PEF) [12]. Moreover, a slight decrease in free-SH contents and H_0_ observed after prolonged treatment (PEF20+US15) may be associated with potential oxidative modifications and/or secondary protein aggregation. A previous study has reported that extended US exposure after PEF can promote the generation of reactive species (H• and •OH), which may contribute to thiol oxidation and disulfide bond formation. In addition, excessive structural rearrangement may promote protein-protein interactions and partial aggregation, potentially masking previously exposed hydrophobic regions [32]. However, these mechanisms remain hypothetical in the present study, as direct measurements of protein oxidation markers were not performed.

### 3.2 Particle size and zeta potential

The particle size and particle size distribution (PSD) of PSPI were significantly influenced by US, PEF, and their sequential treatment (Table 1). Results revealed that US, PEF, and sequential treatment significantly reduced (*p<0.05*) the average particle size of PSPI, with clear changes in PSD curves toward smaller particle diameters. It shows that both the US and PEF significantly disrupt the protein aggregates induced during isolation, which are usually stabilized by electrostatic and non-covalent interactions, including H-bonds and hydrophobic interactions. In individual treatment comparisons, US processing resulted in a significantly higher reduction in particle size than PEF. This particle size reduction is primarily associated with US-induced cavitation. The instant generation and implosive collapse of bubbles (cavitation) induce microjets, high shear forces, and shock waves, which can break down weakened intermolecular interactions and protein aggregates [33]. Earlier, Habib, Singh, Ahmad, Jan, Gupta, Jan and Bashir [8] reported a similar reduction in particle size for pumpkin seed protein isolate after US treatment, in which mechanical effects induced by US cavitation facilitate the disintegration of aggregates and enhance colloidal stability. On the other hand, the observed reduction in PEF is mainly due to electro-deformation and electroporation mechanisms, which induce structural stress that facilitates weakening of intermolecular interactions and partial protein unfolding, resulting in aggregate dissociation [11]. PEF treatment primarily alters molecular conformation rather than inducing strong mechanical shear; its potential to break larger aggregates is lower than that of the US, resulting in a comparatively lower reduction in particle size.

In contrast, the sequential treatment showed the most significant reduction in particle size, suggesting a synergistic effect of both technologies. Pre-treatment with PEF induced structural loosening and partial unfolding of PSPI molecules, exposing charged groups and internal hydrophobic regions, and improving molecular flexibility. These modifications may have decreased aggregate stability and increased susceptibility to US cavitation, which efficiently breaks down aggregates [14]. Synergistic effects of sequential US and PEF treatments have previously been reported for chickpea protein isolate, where reduced particle size were observed with improved dispersion [12]. As shown in Fig. 1B, the control PSPI showed a dominant peak at higher particle size, indicating the large protein aggregates. Individual US and PEF treatments shift the PSD toward smaller diameters, but the PSD remains relatively broad. On the other hand, sequential treatments produced a significant change at the nanoscale, with a single dominant peak exhibiting a narrow PSD. It indicates a particle-homogeneous population, resulting from synergistic structural modification by PEF, followed by US-induced cavitation disruption of aggregates.

The impact of US, PEF, and sequential treatments on the zeta potential of PSPI is presented in Table 1. It is an important factor in electrostatic repulsion, surface charge, and colloidal stability of protein isolates. Results revealed that all individual and sequential treatments significantly increased (*p<0.05*) the negative charge magnitude compared to the control PSPI, showing significant changes in PSPI surface properties. The US treatments showed a higher negative charge than the PEF-treated PSPI. The US induced acoustic cavitation, which generates microjets, high shear forces, transient pressures, and temperatures. This facilitates the breakdown of non-covalent bonds, partial protein unfolding, and disruption of protein aggregates, thereby increasing the exposure of -ve charge groups [22]. Moreover, US treatment also caused a significant reduction in particle size, thereby improving the surface area-to-volume ratio and contributing to the estimation of zeta potential. This improvement in surface charge is usually associated with decreased protein-protein aggregation and improved dispersion stability. Our results align with those of Manzoor, Waseem, Diana, Wang, Ahmed, Ahmed, Ali and An-Zeng [9], who reported a significant increase in negative zeta potential after US treatments.

In contrast, PEF exposure disrupts electrostatic interactions and H-bonds, leading to the redistribution of surface charges and moderate protein structural unfolding [11], [16]. According to the outcomes, PEF is usually less effective than US at generating intense mechanical shearing, which is why it causes comparatively lower structural modification and a smaller increase in -ve zeta potential. The synergistic effect of the sequential treatment resulted in a significant increase in zeta potential, because PEF pretreatment weakens the aggregates and the PSPI structure. Then, the US treatment mechanical shear increased aggregation disruption, protein unfolding, and the exposure of ionizable groups [12], [14].

### 3.3 Intrinsic fluorescence spectra (FS)

**Fig. 2:**
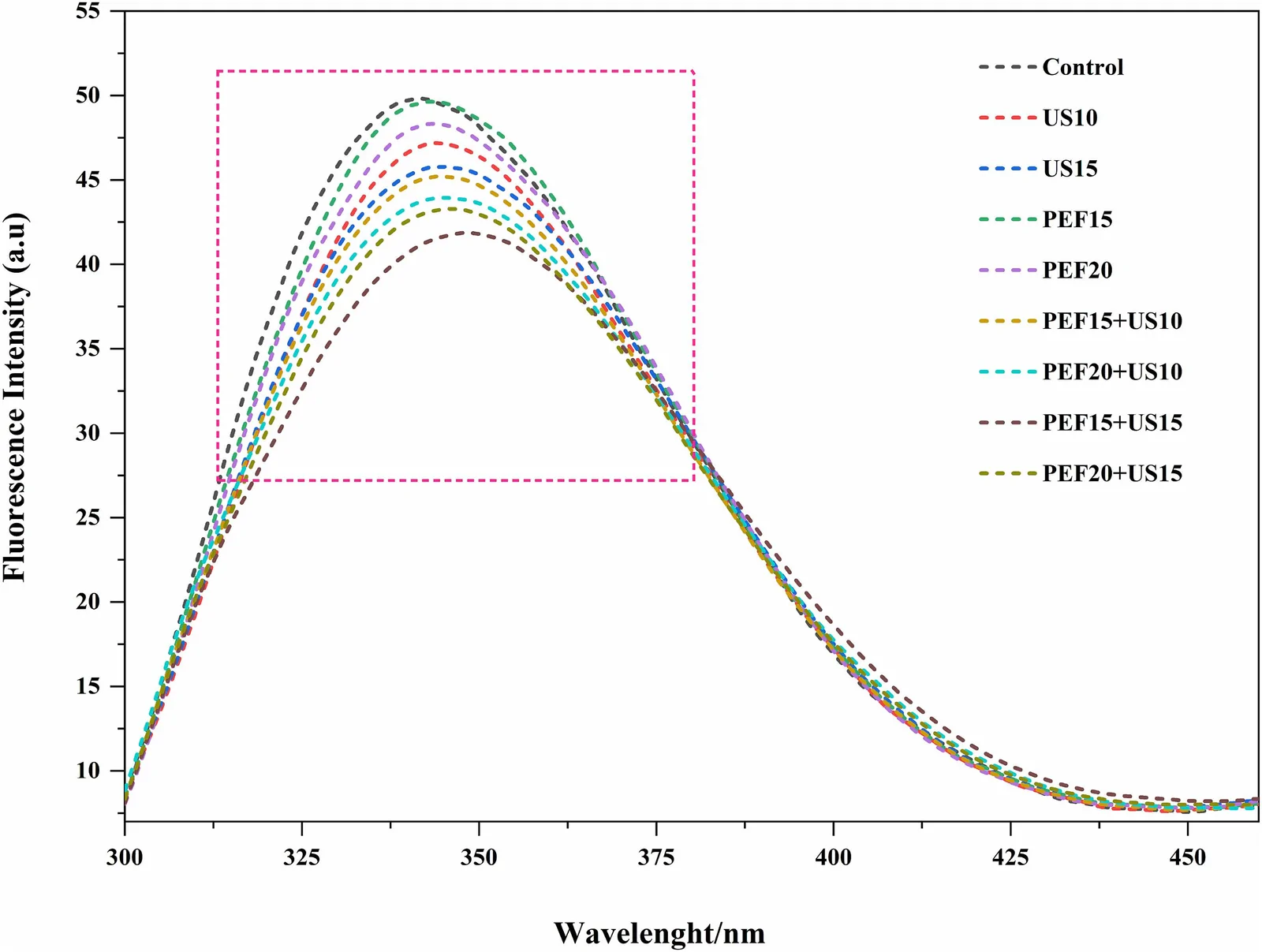
Intrinsic fluorescence spectra of PSPI after the impact of US, PEF, and sequential treatments.

Intrinsic FS was used to analyze conformational changes in PSPI induced by US, PEF, and sequential treatments. Proteins' intrinsic fluorescence usually emerges from aromatic amino acids, such as tryptophan (Trp), whose λ_max and fluorescence intensity (FI) are very sensitive to modifications in microenvironment polarity and the tertiary structure [34]. The control PSPI showed high FI with a characteristic λ_max, indicating that most Trp residues are in a relatively hydrophobic environment. After US, PEF, and sequential treatment, FI decreased significantly (*p<0.05*) compared to the control sample, indicating a significant modification in PSPI conformation (Fig. 2). Upon PEF processing, a decrease can be associated with electroporation, which generates structural changes by disrupting electrostatic forces and H-bonds that stabilize the protein tertiary structure [35]. This type of disruption mainly led to partial protein unfolding, enhancing the exposure of Trp residues to the aqueous phase and facilitating fluorescence quenching by improving their interaction with polar quenchers.

While US treatment induced a more significant decrease in FI than individual PEF, US-induced cavitation produces intense shear forces, localized high pressures and temperatures, which cause protein molecular rearrangements and unfolding [36]. Reduction in FI indicates that the US effectively disrupted the PSPI tertiary structure, thereby increasing the potential for aggregation and aqueous exposure of Trp residues. Moreover, free radicals generated by cavitation may contribute to fluorescence quenching by oxidatively modifying aromatic amino acids [37]. The sequential treatment showed the greatest reduction in FI with a notable shift in λ_max toward longer wavelengths. This red shift indicates that Trp residues were shifted towards a more polar, rather than a non-polar, microenvironment, verifying extensive protein unfolding [12]. The sequential impact of PEF and US intensified structural modification: PEF increased molecular mobility and weakened the protein matrix and the following US induced conformation changes beyond those limits obtained with the treatments alone. These fluorescence shifts align with reported alterations in plant protein isolates after US and PEF treatments, in which decreases in intrinsic FI and shifts in λ_max is associated with better functional properties, including emulsification, foaming, solubility, and digestibility. Partial unfolding and structural weakening expose reactive and hydrophilic groups, which effectively improve techno-functional properties [38].

### 3.4 Secondary structure

**Fig. 3:**
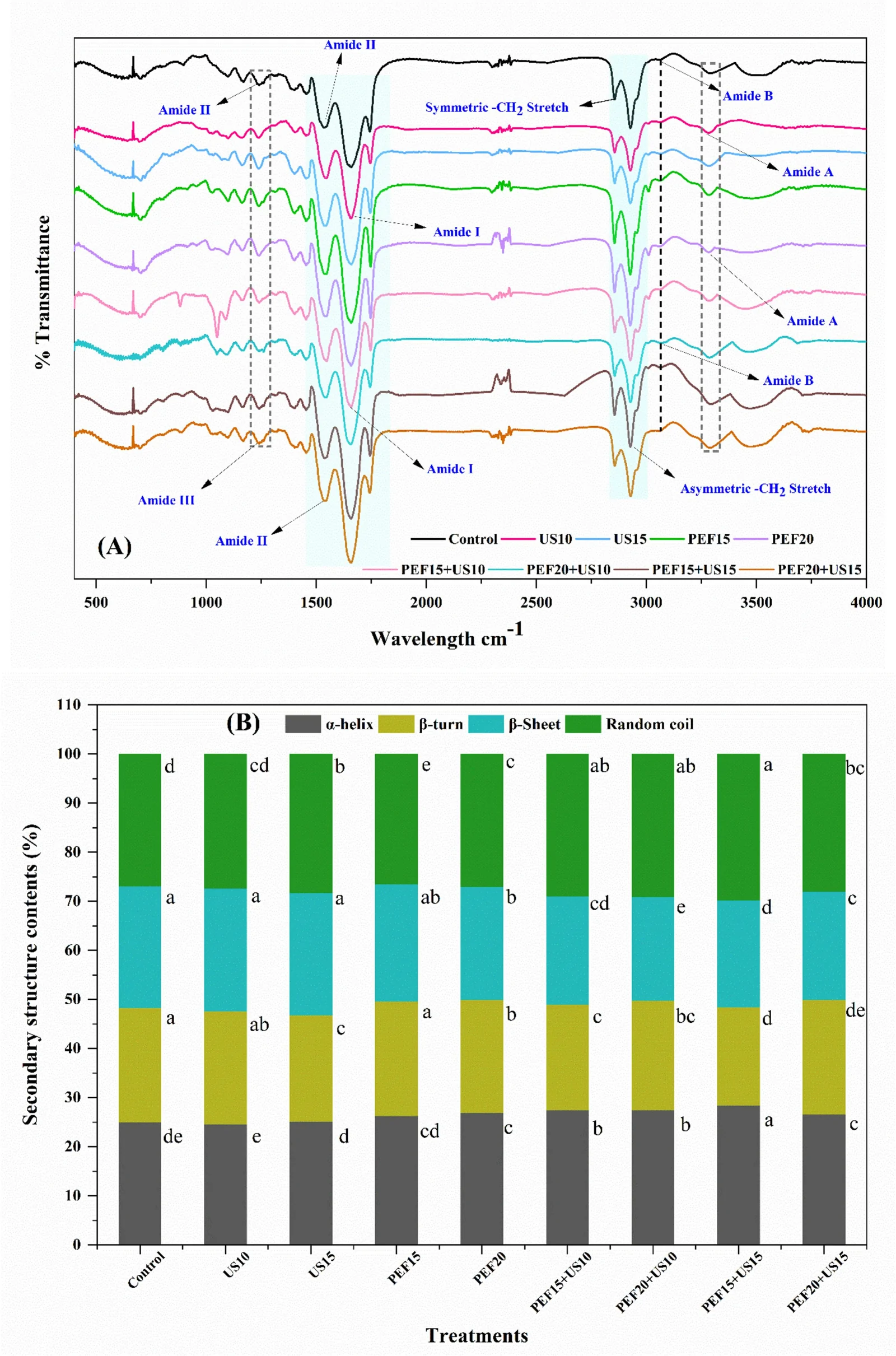
FT-IR spectra **(A)** and Secondary structure contents (%) **(B)** of PSPI after the impact of US, PEF, and sequential treatments.

After US, PEF, and sequential treatments, the FTIR spectra of PSPI showed characteristic vibrational bands, as presented in Fig. 3A. The prominent absorptions belong to Amide A and B (3290-3380 cm^-1^ and 3080-3100 cm^-1^, respectively), which are primarily linked with Fermi resonance and N-H stretching of amide vibrations. It was recorded in all samples, indicating the hydrogen-bonding and backbone-interaction environments. The Amide I, II, and III (1590-1729 cm^-1^, 1485-1585 cm^-1^, and 1200-1285 cm^-1^, respectively) bands, which occur from C-N stretching, C=O stretching, and N-H bending vibrations of the peptide backbone, are the basis for resolving the secondary structure analysis [39]. Quantitative deconvolution of the Amide I region indicated that specific modifications in PSPI secondary structural components occurred among all processing treatments, as shown in Fig. 3B. The US, PEF, and sequential treatment samples showed a notable increase in % transmittance and a shift in the peak of the Amide I, II, and III regions compared to the control sample. It indicates modifications in protein conformational arrangement and backbone H-bonding. In contrast, Amide A and B show a relative intensity shift indicating alteration in H-bond networks and N-H bond strength after processing, as shown in Fig. 3A.

Amide I curve fitting and deconvolution state the structural components of PSPI: random coil (1640-1650 cm^-1^), α-helix (1650-1660 cm^-1^), and *β*-turn (1665-1686 cm^-1^). Results revealed that US, PEF, and sequential treatments significantly (*p<0.05*) increased random coil and α-helix contents, while decreasing *β*-turn and *β*-sheet structures. The rise in random coil and α-helix content indicates a shift toward less ordered, more flexible conformations, due to the breakdown of H-bonds that stabilize *β*-sheet structures, facilitating chain mobility and protein unfolding [40]. While *β*-sheets are typically associated with compact protein or aggregated conformations, their reduction indicates enhanced molecular dispersion and decreased aggregation. The sequential treatment resulted in a significant modification of the secondary structure. Firstly, PEF generates electroporation, weakening electrostatic forces and H-bonds and inducing polarization of charged amino acid residues [3]. Then, US-induced acoustic cavitation, which generates transiently high temperatures, microturbulence, and high shear forces, can destabilize or modify ordered proteins, facilitating their unfolding [36].

The amide II region belongs to C-N stretching and N-H bending vibrations and showed an increase in % transmittance after processing. It is revealing that alterations in the H-bonding pattern and the peptide-bond environment further support secondary-structure rearrangement and protein unfolding. Similarly, in the amide III region, modifications were observed, which are sensitive to side-chain interactions and backbone conformation. The band shift and increase in % transmittance in this region indicate modification in backbone flexibility and dihedral angles; these changes are consistent with an increase in random coil content. This structural loosening (increased exposure of reactive groups) is also associated with increased functional attributes such as emulsifying properties and solubility [30], [41]. Significant changes were also noticed in amide A and B regions. The changes in the amide B region due to asymmetric CH stretching also suggest changed hydrophobic interactions and rearrangement of aliphatic side chains. The amide A band shift and increased intensity are associated with N-H stretching vibrations, indicating weakened H-bonding and resulting structural flexibility. In conclusion, protein modification via controlled unfolding is desirable, as it enhances structural flexibility while maintaining protein backbone integrity. These observed structural modifications after US and PEF align with the literature, which reports increased techno-functional properties of protein isolates [11], [22], [30].

### 3.5 SEM

**Fig. 4:**
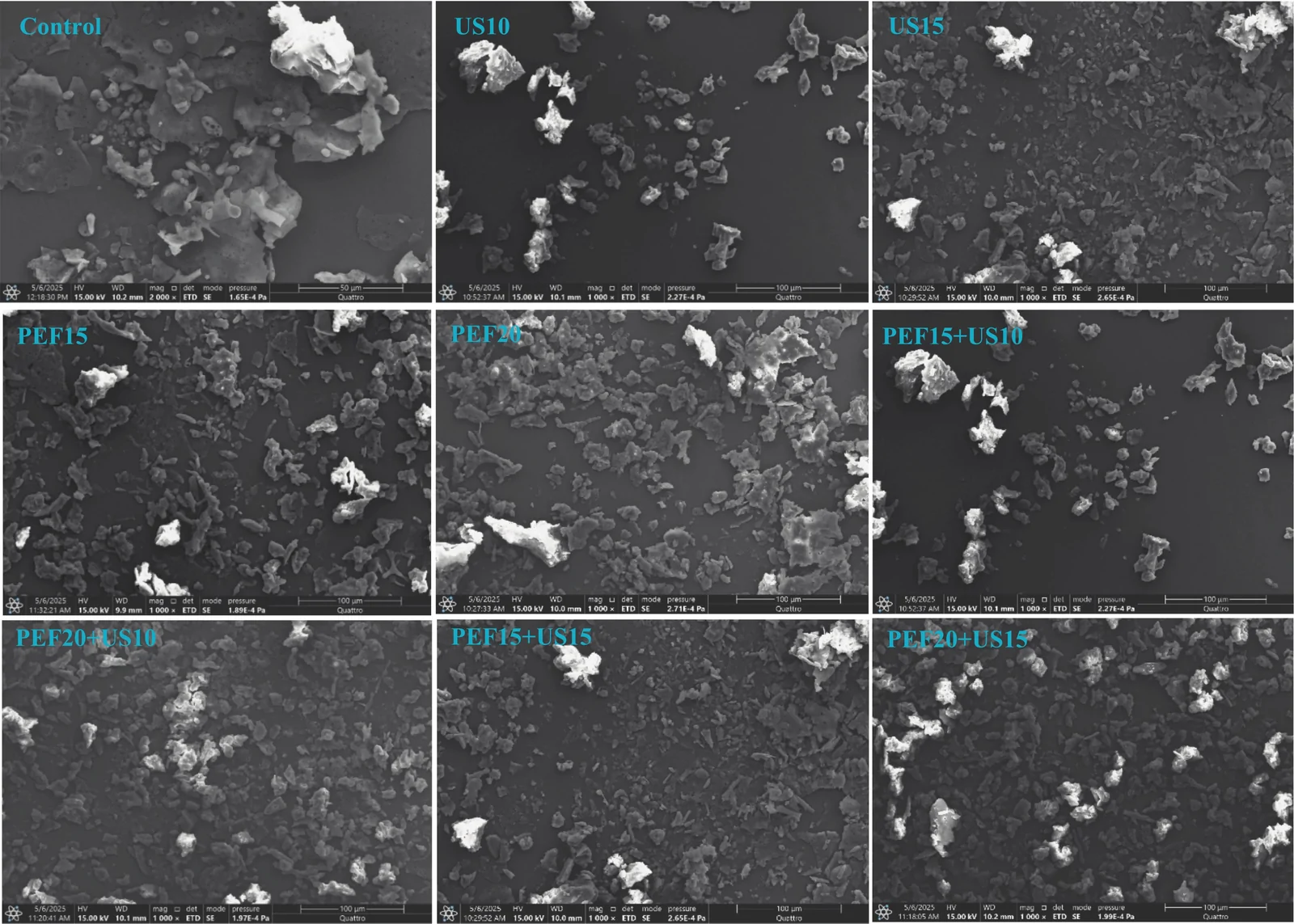
SEM images of PSPI after US, PEF, and sequential treatments.

The US, PEF, and sequential treatment induced microstructural modifications in PSPI, as observed by SEM (Fig. 4). Specific changes in particle size, structural integrity, and surface morphology were observed among treatments, with the sequential treatment showing the most significant alterations. The control sample SEM images showed large, irregularly shaped compact aggregates with smooth, dense surfaces, indicating limited exposure of internal structures and strong intermolecular binding. This compact morphology of PSPI is maintained by H-bonding and hydrophobic interactions. PEF-treated SEM images showed the pores, fragmented structure, and partial disruption of compact aggregates. The modification associated with PEF-induced polarization and electroporation facilitates structural loosening and weakening of non-covalent interactions [42]. Earlier, microstructural breakdown of quinoa protein isolates has been reported, characterized by decreased particle cohesion and increased matrix porosity [41]. US treatment exhibited greater disruption of aggregates, reductions in particle size, pores, and roughness compared with PEF. The US-induced cavitation generates microjets, shear forces, and shock waves, disrupting protein aggregates and facilitating redistribution and protein unfolding, as indicated by changes in particle size and surface erosion [43]. The SEM images revealed that US treatment altered the surface morphology and microstructure of pumpkin seed protein isolate particles, resulting in a more fragmented and porous structure. These morphological changes may be associated with the enhanced solubility and digestibility observed in this study [8]; however, protein unfolding and conformational flexibility were primarily inferred from complementary structural analyses rather than SEM observations alone.

The sequential treatment showed the most significant microstructural modification, with porous, highly fragmented, and loosely arranged structures with noticeably increased roughness and decreased particle size. In a sequential action, PEF-generated electroporation followed by US cavitation enhances protein disintegration, thereby causing significant disruption of aggregates [32]. These microstructural modifications are associated with secondary structural alterations: an increase in random coil and α-helix, and a reduction in *β*-turns and *β*-sheet structures, indicating loss of ordered structures and protein unfolding. The disruption of *β*-sheet domains is associated with increased rigidity and protein aggregation, as evidenced by aggregate compactness and a decreased particle size. In contrast, random coil (increased) improved molecular flexibility, which facilitates fragmentation, as observed in the SEM images in Fig. 4.

### 3.6 XRD

**Fig. 5:**
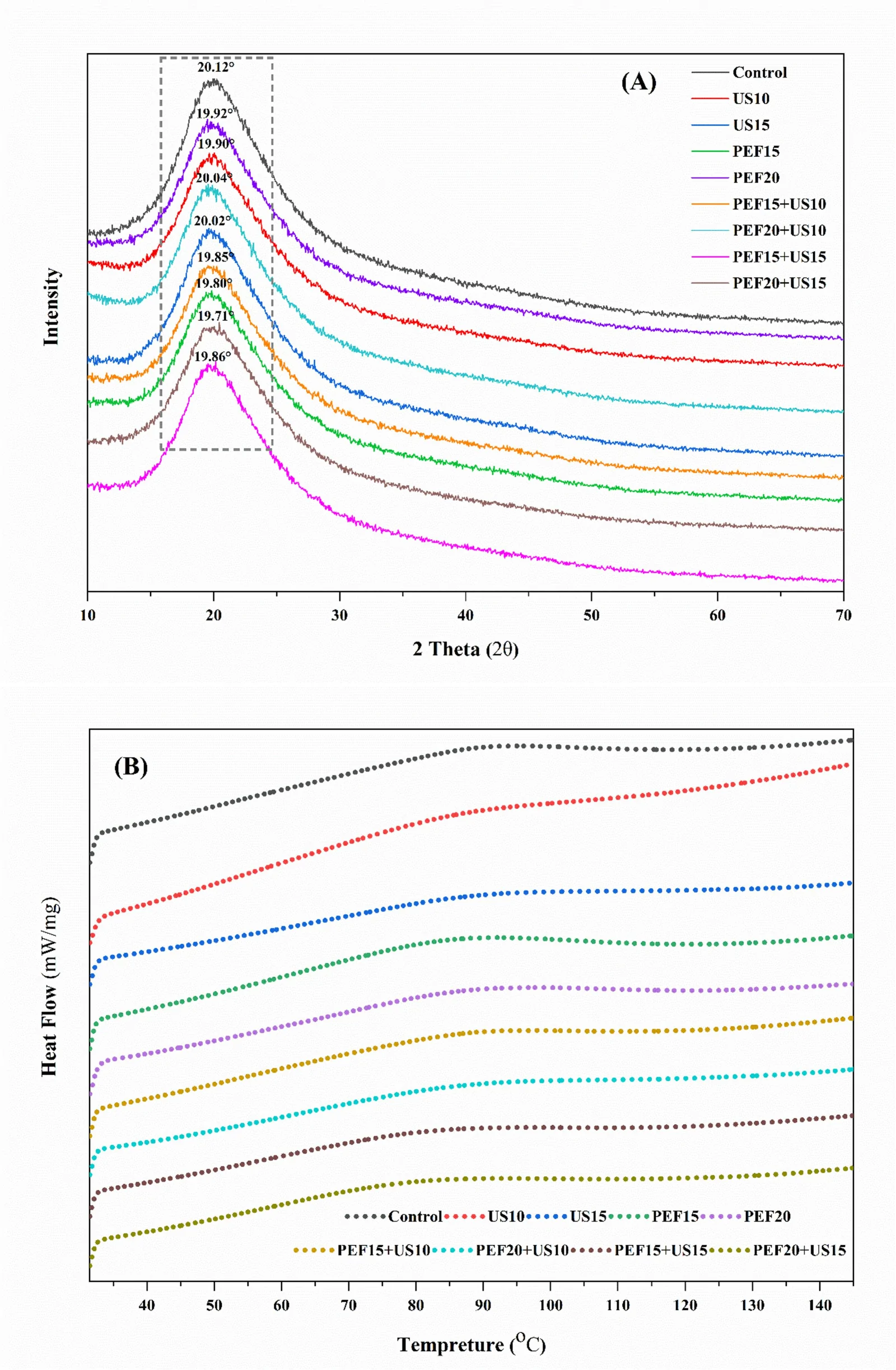
X-ray diffraction (XRD) spectra **(A)**, and Differential scanning calorimetry (DSC) thermograms **(B)** of PSPI after the impact of US, PEF, and sequential treatments.

The XRD patterns of PSPI showed clear structural modifications after US, PEF, and sequential treatments. The control PSPI sample showed a broad diffraction peak at 2θ≈20.11°, which indicates that the protein has a semi-crystalline structure dominated by *β*-sheet arrangements within an amorphous matrix [44]. A significant decrease in peak intensity was observed across all processing treatments, in the following order: sequential treatments > US > PEF > Control, indicating a progressive breakdown of the crystalline regions. Among individual treatments, US processing showed a significant reduction in peak intensity compared with PEF, suggesting an intense influence on long-range PSPI molecular order. In contrast, the sequential treatment showed the lowest diffraction peak intensity with a slight shift from 20.11° to 19.71°, indicating changes in protein chain packing and interplanar spacing (Fig. 5A).

The reduction in peak intensity after processing indicates a decline in crystallinity within the protein matrix and an improvement in amorphous regions. US processing mainly generates cavitation, which induces shear forces and localized high pressures and temperatures that weaken intermolecular bonds and disrupt H-bonds, thereby stabilizing ordered protein regions [8]. On the other hand, PEF-induced polarization by electroporation reorients charged side chains and induces partial unfolding, which moderately disrupts the crystalline domains without excessive mechanical breakdown [4]. Earlier, a comparative study reported that PEF and US induced conformational changes and a loss of relative crystallinity in fava bean protein isolate, with a decrease in peak intensity associated with reduced structural order and particle size [22].

The sequential effect is associated with the sequential action of electrical (PEF) and mechanical forces (US). Firstly, PEF increased molecular mobility and pre-unfolding, thereby enhancing the susceptibility of protein aggregates to US, resulting in greater structural disruption [12]. After sequential treatments, the observed red-shifted 2θ values indicate that an expansion of lattice spacing aligns with loosening of molecular packing and protein unfolding. Conclusively, the significant peak shift and reduction in diffraction intensity after sequential PSPI treatment revealed a substantial loss of crystalline domains. These structural changes are mainly associated with improved functional properties and highlight the ability of these technologies to modify PSPI [14].

### 3.7 DSC

DSC was used to determine the impact of US, PEF, and sequential treatment on the thermal properties of PSPI, as shown in the thermograms in Fig. 5B and in the tabulated data of onset/offset/denaturation temperatures and enthalpy change in Table 2. The control PSPI sample showed a clear endothermic transition with a T*_d_* of 89℃, characteristic of the cooperative unfolding of protein structures stabilized by disulfide linkages, hydrophobic interactions, and H-bonding. After US, PEF, and sequential treatments, a significant (*p<0.05*) reduction in ΔH was observed compared with the control sample, indicating a substantial modification in protein conformational order before thermal scanning. The ΔH reduction indicates that some energy-intensive binding remained disrupted during heating, showing partial protein unfolding and decreased PSPI structural compactness. A similar reduction in ΔH following US and PEF treatment has been reported for soy and pea protein isolates, due to the loss of native tertiary structure and weakened intramolecular interactions [5], [8].

In addition to enthalpy changes, US, PEF, and sequential treatments affect the T*_d_*. A minor but significant decline in T*_d_* was observed for individual US- and PEF-treated PSPI. The changes in the PEF-treated sample were due to disruption of electrostatic bindings and dipole realignment induced by the electric field, which stabilize the protein domain [5], [41]. Earlier, De Gol, De Ridder, Zwietering, den Besten and Beyrer [5] reported a similar decrease in ΔH and T*_d_* of pea protein after PEF treatment. In contrast, US-induced acoustic cavitation generates localized transient thermal hotspots, elevated pressures, and intense shear forces, which facilitate exposure of hydrophobic regions, dissociate protein aggregates, and promote irreversible unfolding, resulting in reduced unfolding cooperativity and thermal stability [8], [22]. Our results align with those of Habib, Singh, Ahmad, Jan, Gupta, Jan and Bashir [8], who reported a decrease in thermal stability after US treatment of pumpkin seed protein isolate. Notably, the sequential treatment (PEF15+US15) showed the lowest ΔH (100.27 J/g) and T*_d_* (81.99℃) values among all US, PEF, and control samples. During this synergistic effect, PEF pre-treatment increased molecular flexibility and partially destabilized molecules, thereby increasing their susceptibility to US cavitation. It intensifies the disruption of ordered domains and non-covalent interactions, thereby decreasing thermal stability [12], [22], [45]. Conclusively, the decreases in T*_d_* and ΔH indicate that US, PEF, and sequential treatments significantly affect the PSPI structural confirmation and thermal stability.

### 3.8 Solubility

**Fig. 6:**
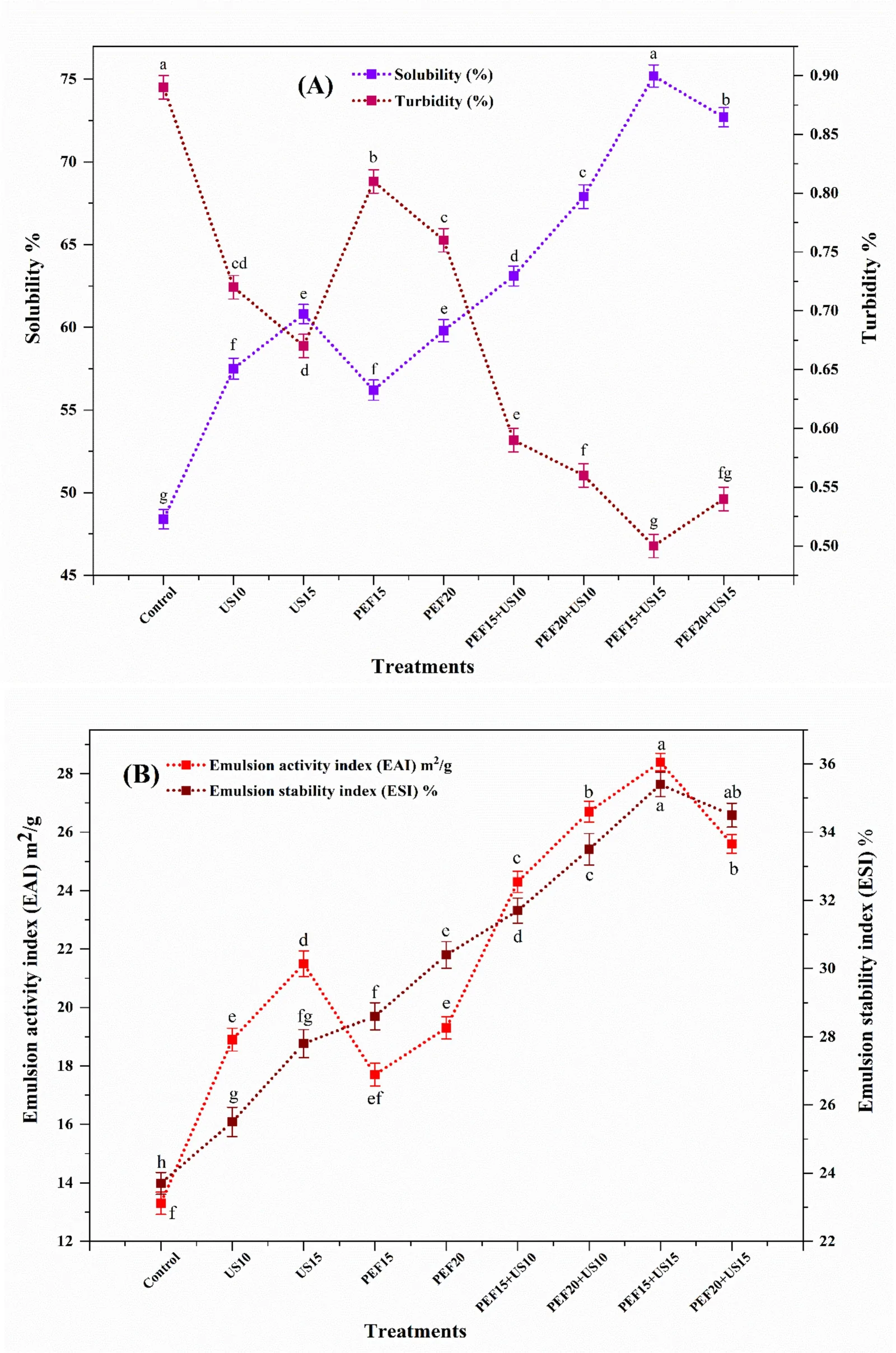
Solubility and Turbidity **(A)**, and Emulsion activity and Emulsion stability index **(B)** of PSPI after the impact of US, PEF, and sequential treatments. All the results (n = 3) are presented as mean ± S.D., while the different alphabets (a-g) above the bars show the significant difference (*p* < 0.05) between treatments and control (Tukey’s HSD).

The solubility of PSPI was significantly increased (*p<0.05*) after US, PEF, and their sequential impact compared with the control (Fig. 6A). This significant increase in solubility indicates that non-thermal processing effectively altered PSPI structures, thereby increasing their water interaction. Results showed that individual US treatment had higher solubility (60.8±0.58 %) than PEF (58.8±0.63 %); in contrast, the sequential treatment (PEF15+US15) showed the most significant increase (75.2±0.67 %). The US-induced acoustic cavitation produces microstreaming and localized high-shear forces that expose buried hydrophilic groups, break non-covalent bonds, and partially unfold molecules, thereby improving protein interactions with water [22], [46]. Earlier, Gao, Zha, Yang, Rao and Chen [46] reported that US treatment increased the solubility of pea protein isolate, with unfolding and enhanced dispersion without significant aggregation.

In PEF-treated samples, solubility significantly increased but was less than that of US-treated samples. This increase is due to electric-field-induced polarization and electroporation of PSPI molecules, which modify tertiary and secondary structures and weaken intermolecular bindings [41]. Also, the PEF induces lower mechanical shear compared with US. Its potential to break protein aggregates is relatively lower, which explains the lower improvement in solubility [22]. A similar increase in solubility has also been reported in PEF-treated quinoa protein [41] and faba bean protein isolate [22]. Solubility increases after the sequential treatment, possibly due to the synergistic impact of electric fields and US-induced cavitation, which facilitates more significant structural rearrangement. PEF weakens the intermolecular interactions of PSPI as a precondition; later, US induces dispersion and unfolding. A similar synergistic impact of sequential PEF+US for the improvement of solubility in chickpea protein isolate was reported by Lai, Chen, Nie, Guo, Manzoor, Huang, Li, Lin, Zeng and Wang [12].

### 3.9 Turbidity

Turbidity decreased, whereas solubility increased; the control PSPI sample showed higher turbidity (0.89±0.01 %), while US, PEF, and their sequential treatments exhibited significantly lower (*p<0.05*) turbidity (Fig. 6A). Individual US-treated samples showed lower turbidity than PEF-treated samples, while the sequential treatment (PEF15+US15) demonstrated the most significant decrease (0.5±0.01 %) among all samples. Generally, a decrease in turbidity indicates the breakdown of protein insoluble particles or aggregates. The greater reduction in turbidity in US-treated samples than in PEF-treated samples is in line with the better solubility observed. Strong US-induced shear forces can disrupt aggregates into smaller particles, thereby dispersing more uniformly and limiting light scattering, although this occurs when PSPIs are partially unfolded. Notably, the balance between deagglomeration and protein unfolding likely accounts for the decrease in turbidity observed in sonicated samples [9]. Our results align with those of Habib, Singh, Ahmad, Jan, Gupta, Jan and Bashir [8] for pumpkin seed protein isolate, further stating that the effect of US on decreasing turbidity is associated with aggregates breakdown and enhanced surface charge, thereby improving colloidal stability.

PEF reduced turbidity due to structural rearrangements induced by the electric field, which decrease protein aggregate size and weaken intermolecular bindings. At 20 kV, PEF-induced polarization redistributes surface charges and causes partial unfolding, thereby improving electrostatic repulsion and limiting reaggregation [16]. Our results align with those of Wang, Guo, Yang, Huang, Wang, Li, Lin, Sheng, Zeng and Teng [11], who described a similar reduction in turbidity of soybean protein isolate after PEF treatment at 10 kV and 15 kV. The sequential treatments showed the most significant reduction in turbidity, indicating a synergistic effect between US cavitation and electric-field exposure. Firstly, PEF disrupts intermolecular interactions, making protein aggregates more susceptible to US shear forces, thereby enhancing dispersion uniformity and extensive deagglomeration. These synergistic decreases in turbidity following sequential treatments are associated with improved protein functional properties [47]. Notably, a slight increase in turbidity was observed after prolonged sequential treatment (PEF20+US15). It reaggregates unfolded proteins via hydrophobic binding, forming small soluble aggregates that slightly enhance light scattering without exceeding control turbidity levels.

### 3.10 EAI and ESI

The impact of US, PEF, and their sequential treatments on the EAI and ESI of PSPI is shown in Fig. 6B. Results showed that US, PEF, and sequential treatments significantly increased (*p<0.05*) the EAI and ESI of PSPI compared to the control sample (13.3±0.38 m^2^/g and 23.7±0.31 %), respectively. Individual US treatment significantly increased EAI compared with PEF treatment; in ESI, PEF treatment showed better results. Overall, the sequential treatment, especially PEF15+US15, showed the greatest increases in EAI (28.4±0.28 m^2^/g) and ESI (35.4±0.38 %) compared with all other treatments. EAI improvement shows that the treated PSPI rapidly absorbs at the interface (oil-water) and produces smaller oil droplets. US-induced acoustic cavitation produces transient hot spots, microturbulence, and high shear force, which break non-covalent bonds within aggregates. It exposes hydrophobic domains and flexible protein molecules, leading to rapid interface adsorption and improved emulsion formation [22]. In PEF treatment, the electric field induces conformational rearrangements and polarization in protein molecules by disrupting H-bonds and electrostatic bindings. As a result, the redistribution of hydrophobic and charged residues, along with moderate unfolding, increases interfacial activity and solubility [48]. In PEF treatment, the emulsifying properties are comparatively lower than in the US, because mechanical shear is absent, limiting the level of structural breakdown. Earlier, Zhang, Li, Zheng, Wen and Wang [41] reported a significant increase in EAI and ESI of PEF-treated quinoa protein isolate, while Gulzar, Martín-Belloso and Soliva-Fortuny [22] reported a significant rise in US-treated faba bean protein isolate. A more significant increase in emulsifying properties was observed following sequential treatment due to a synergistic relationship between mechanical and electrical impact. Pretreatment with PEF first reduces aggregate stability and disrupts internal interactions, thereby increasing the PSPI molecule's susceptibility to US cavitation. This successive action facilitates reduced particle size, extensive unfolding, and improved interfacial properties, thereby enhancing emulsion-formation ability [45]. A slight decline in EAI and ESI was observed at the PEF20+US15 treatment, due to aggregation and excessive unfolding due to overexposure to cavitational and electric stresses. It facilitates re-aggregation and intermolecular binding and decreases flexibility, thereby reducing interface (oil-water) adsorption.

### 3.11 FC and FS

**Fig. 7:**
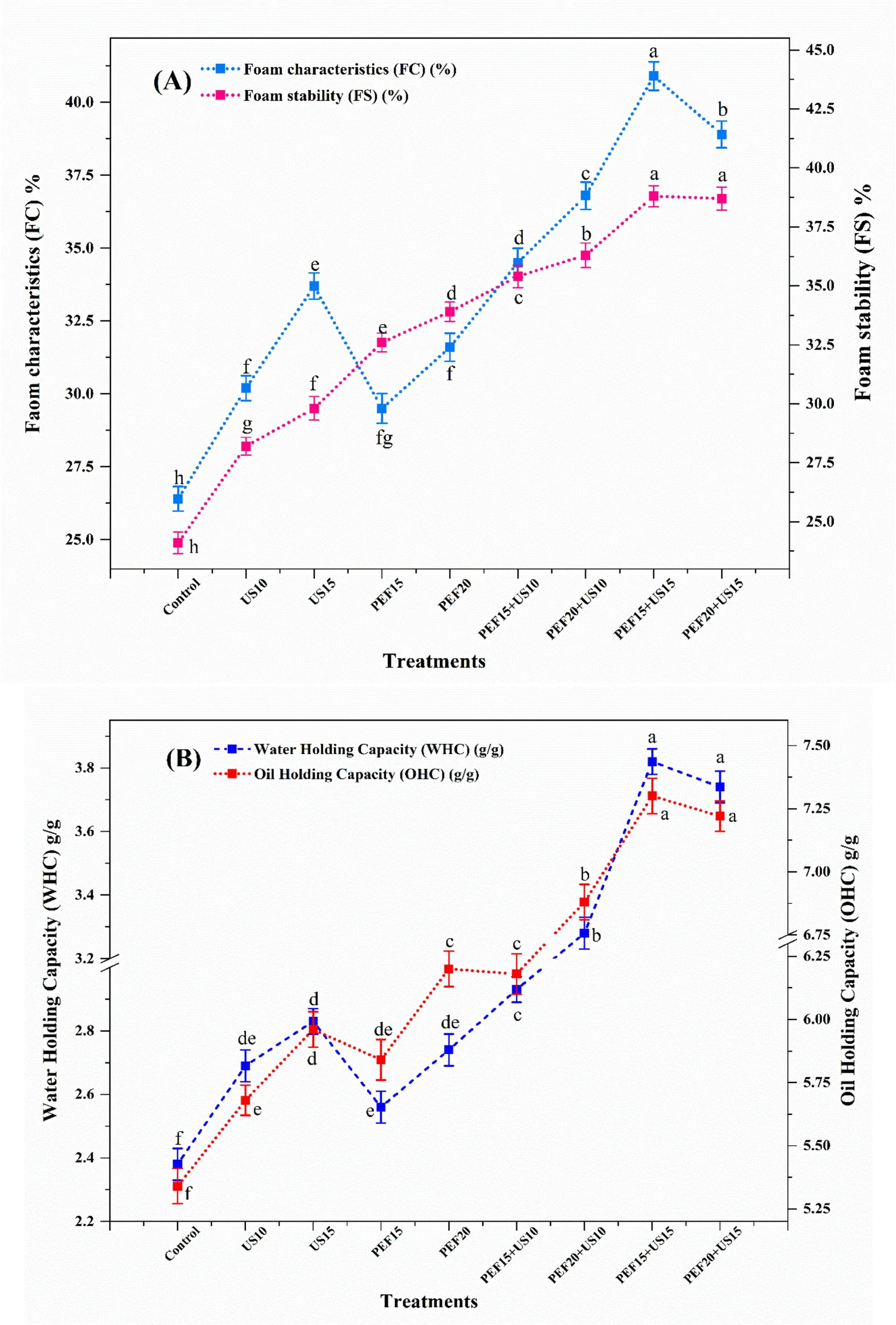
Foam characteristics and Foam stability **(A)**, Water-holding capacity and Oil-holding capacity **(B)** of PSPI after the impact of US, PEF, and sequential treatments. All the results (n = 3) are presented as mean ± S.D., while the different alphabets (a-g) above the bars show the significant difference (*p* < 0.05) between treatments and control (Tukey’s HSD).

The FC and FS of PSPIs were significantly increased (*p<0.05*) by US, PEF, and sequential treatments compared to the control sample, as shown in Fig. 7A. Results revealed that these non-thermal modification techniques improved the interfacial activity of PSPI, with the most significant increase observed in the sequential treatment (PEF15+US15) compared with individual PEF, US, and untreated samples. Among the individual US and PEF treatments, the US -treated samples showed better results in improving FC and FS than the PEF-treated samples. US is known to generate acoustic cavitation, transient hot spots, and microstreaming, and induce high shear forces, which partially unfold proteins, exposing flexible peptide segments and hydrophobic amino acid residues, thereby facilitating rapid adsorption at the PSPI interface (air-water) [49]. Thus, the US-treated PSPI showed better air incorporation and film formation around air bubbles. Our results align with earlier studies that documented increased FC and FS in chickpea [49] and in sunflower meal protein isolate [23] after US treatment due to increased H_0_ and reduced particle size.

The increase in the PEF-treated sample associated with the electric field and electroporation induced conformational rearrangements of PSPI molecules. These electric fields break non-covalent bonds, such as electrostatic forces and H-bonds, resulting in enhanced molecular flexibility and partial unfolding [48]. These structural modifications increased the protein arrangement at the interface and diffusivity, resulting in improved foam formation. As a result, PEF induced less mechanical shear than US treatment, with a significantly lower particle size reduction (Table 1) and a lower degree of protein unfolding, indicating its relatively lower impact on FC and FS. The sequential (PEF+US) treatments improved foaming properties by promoting optimal partial unfolding and limiting over-aggregation, thereby inducing a stable protein conformation with better interfacial activity. A similar synergistic effect of sequential PEF+US treatment has been reported for chickpea protein isolate [12] to improve FC. A decrease in particle size increases interface packing density, and higher solubility facilitates the migration of protein molecules to the water-air interface, producing an elastic and cohesive interfacial film [50]. Moreover, hydrophobic groups (exposed) support protein-protein binding within the foam lamella, decreasing bubble drainage and coalescence, resulting in improved FS.

### 3.12 WHC and OHC

The OHC and WHC of PSPIs were significantly increased (*p<0.05*) by US, PEF, and sequential treatments compared to the control (Fig. 7B). Individual US showed a significant increase in WHC compared to PEF-treated samples, whereas in OHC, PEF-treated samples showed better results. In contrast, the sequential treatment PEF15+US15 exhibited the highest improvement, followed by PEF20+US15. WHC reflects the protein's capacity to retain and entrap water within its structural matrix, which is linked to the exposure of hydrophilic groups, protein porosity, and hydration. In the PEF-treated sample, the increase in WHC can be associated with structural modifications induced by electroporation, which partially unfold protein molecules and break non-covalent bindings. These protein unfoldings increase the availability of polar amino acid residues that bind water, thereby improving hydration capacity [51]. While US treatment primarily improves WHC by inducing microjets, generating shear forces, and inducing acoustic cavitation, which decrease aggregation and increase surface area [10]. Moreover, excessive cavitation in the US-treated sample might lead to structural collapse or partial reaggregation, which could account for the relatively lower WHC compared with PEF-treated samples. During sequential treatment, the synergistic first PEF facilitates loosening of the protein network and molecular unfolding, making the PSPI more susceptible to acoustic cavitation. This disruption leads to a more porous, open protein matrix, thereby improving water entrapment [32]. Moreover, this integrated processing improved the protein-water interactions by reducing particle size and increasing solubility, as reported in our study.

OHC is a parameter that indicates the capacity to entrap oil via hydrophobic binding and is vital for texture development and flavor retention in food products. PEF treatment improved OHC by exposing previously buried amino acid residues in the native protein. Also, the electric field and electroporation-induced conformational modifications enhance protein flexibility and facilitate interactions of higher lipid molecules [42]. In the US-treated sample, an increase in OHC was observed due to the disruption of aggregates and a reduction in particle size, thereby increasing the surface area for oil binding. In the US-treated sample, OHC is comparatively lower due to excessive mechanical stress resulting in decreased structural integrity and partial protein denaturation [9]. A significant increase in OHC following sequential treatment reflects a synergistic improvement in hydrophobic interactions [32]. PEF treatment exposes hydrophobic regions and improves flexibility, while US treatment prevents aggregation and increases particle dispersion. Collectively, it promotes oil entrapment within interstitial spaces created between well-dispersed, unfolded protein particles, thereby maximizing oil retention capacity.

### 3.13 Antioxidant properties

**Fig. 8:**
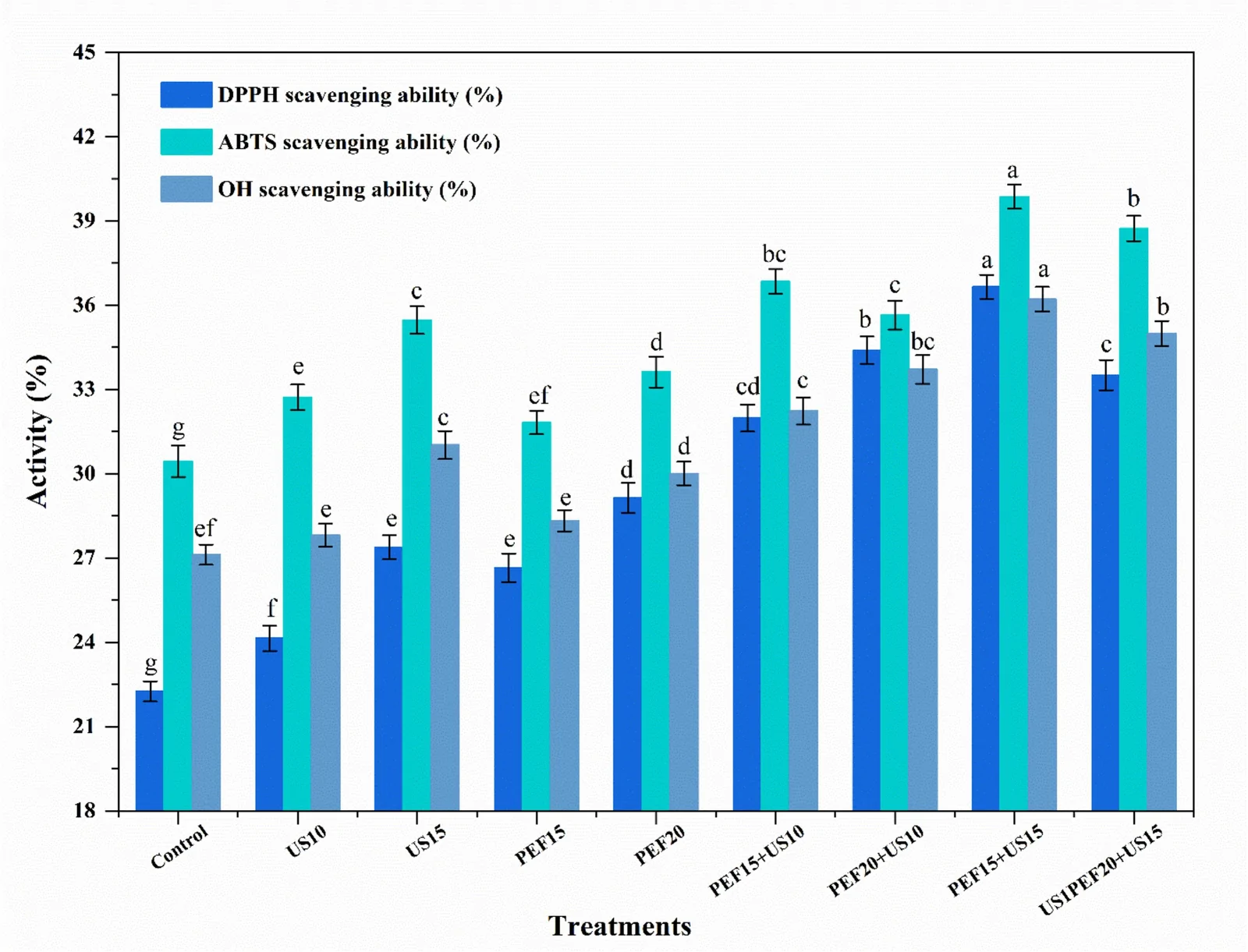
Antioxidant properties of PSPI after the impact of US, PEF, and sequential treatments. All the results (n = 3) are presented as mean ± S.D., while the different alphabets (a-g) above the bars show the significant difference (*p* < 0.05) between treatments and control (Tukey’s HSD).

The US, PEF, and their sequential treatments significantly (*p<0.05*) enhanced the ABTS, DPPH, and •OH scavenging activities of PSPI compared to the control sample, as shown in Fig. 8. Results revealed that individual US treatment yielded better ABTS and •OH scavenging activities than PEF treatment, whereas for DPPH, PEF-treated samples showed better results. Notably, the sequential treatment (PEF15+US15) yielded the highest •OH, ABTS, and DPPH scavenging activities compared to all other treatments, demonstrating a synergistic impact of these technologies on the antioxidant abilities of PSPI.

The overall improvement in all antioxidant properties is associated with treatment-induced physicochemical and structural alterations in PSPI. The antioxidant abilities of proteins are heavily based on amino acid composition, molecular conformation, and the availability of reactive side chains capable of donating hydrogen or electron atoms. US processing generates acoustic cavitation, which induces shear forces, localized high pressures, and elevated temperatures, all of which are effective at disrupting protein tertiary and secondary structures [52]. These may facilitate partial protein unfolding, potentially exposing amino acid residues and hydrophobic regions that could contribute to electron transfer, hydrogen atom donation, and metal-chelating activities [52], [53]. However, the specific amino acid residues involved were not identified in the present study, and further analyses are required to verify this mechanism. This mechanism accounts for the better results of US treatment in the ABTS and OH assays of PSPI, which are based on single-electron and H-atom transfer pathways. In contrast, PEF treatment disrupts non-covalent interactions, including hydrophobic interactions and H-bonds, by inducing electroporation and electric-field polarization, leading to partial protein unfolding and enhanced exposure of buried amino acids [45]. It further alters electrostatic interactions and loosens protein structure, thereby improving protein surface charge and flexibility, ultimately enhancing its ability to scavenge OH radicals [48].

In a recent study by Zhang, Zhang, Huang, Li, Wang, Zeng and Gao [54], it was shown that PEF can regulate protein aggregation and induce structural rearrangement, potentially exposing antioxidant-active groups. Therefore, the enhanced antioxidant activity observed after PEF treatment may be attributed to PEF-induced conformational changes and increased accessibility of reactive sites.

The most significant increase in the antioxidant properties of PSPI recorded under the sequential treatment (PEF+US) demonstrates a pronounced synergistic effect. Initially, PEF acts as a protein structural preconditioning to improve its susceptibility to US cavitation. Then the US treatment escalates molecular breakdown, facilitating reduced aggregation, greater unfolding, and enhanced surface area. This integrated impact improved mass transfer efficiency and led to more useful interaction between radical species and PSPI [14]. The observed increase in OH scavenging ability suggests that the treatments improved the accessibility of side-chain reactive amino acids and metal ion chelation potential. OH radicals are more reactive and require an effective quenching mechanism; thus, the improved OH scavenging ability may lead to the induction of low-molecular-weight peptides and the exposure of functional residues with enhanced antioxidant activity [48]. PEF-induced structural modifications and US-assisted fragmentation may together yield these effects.

### 3.14 IVPD

The US, PEF, and sequential treatments significantly (*p<0.05*) increased the IVPD of PSPI compared with the control sample (82.23±0.58%), as presented in Table 1. Among all, the sequential treatment (PEF15+US15) showed the most significant increase (89.02±0.38%) in IVPD, due to the synergistic effect of US and PEF. PEF increased the IVPD via electroporation, resulting in structural modifications in the protein molecules. It induces conformational rearrangements and permanent or transient pores by breaking non-covalent interactions, including electrostatic forces and H-bonds, thereby improving enzyme accessibility to peptide bonds [55]. In contrast, US-induced cavitation generates shear forces, localized high pressure, and elevated temperatures, which facilitate aggregate disruption, protein unfolding, and particle size reduction. These modifications further exposed cleavage sites and buried hydrophobic residues, thereby promoting protease penetration during digestion [56]. Our results align with previous studies reporting increases in IVPD after PEF treatment of rice bran protein [42] and US-treated buckwheat protein isolate [56]. Notably, the sequential treatment showed the highest digestibility, due to a synergistic mechanism that intensifies molecular fragmentation and unfolding, producing a protein structure more prone to enzymatic hydrolysis [57]. Our structural analysis results mechanistically support the increase in IVPD as sequential treatment increased random coil and α-helix structures while decreasing *β*-turn and *β*-sheet structures. *β*-sheet due to extensive H-bonding, known to form protease-resistant compact structures. Their reduction and increase indicate improved structural flexibility and protein unfolding, thereby directly increasing digestibility [58].

### 3.15 Electro-acoustic coupling: Mechanistic pathway to modify the properties of PSPI

**Fig. 9:**
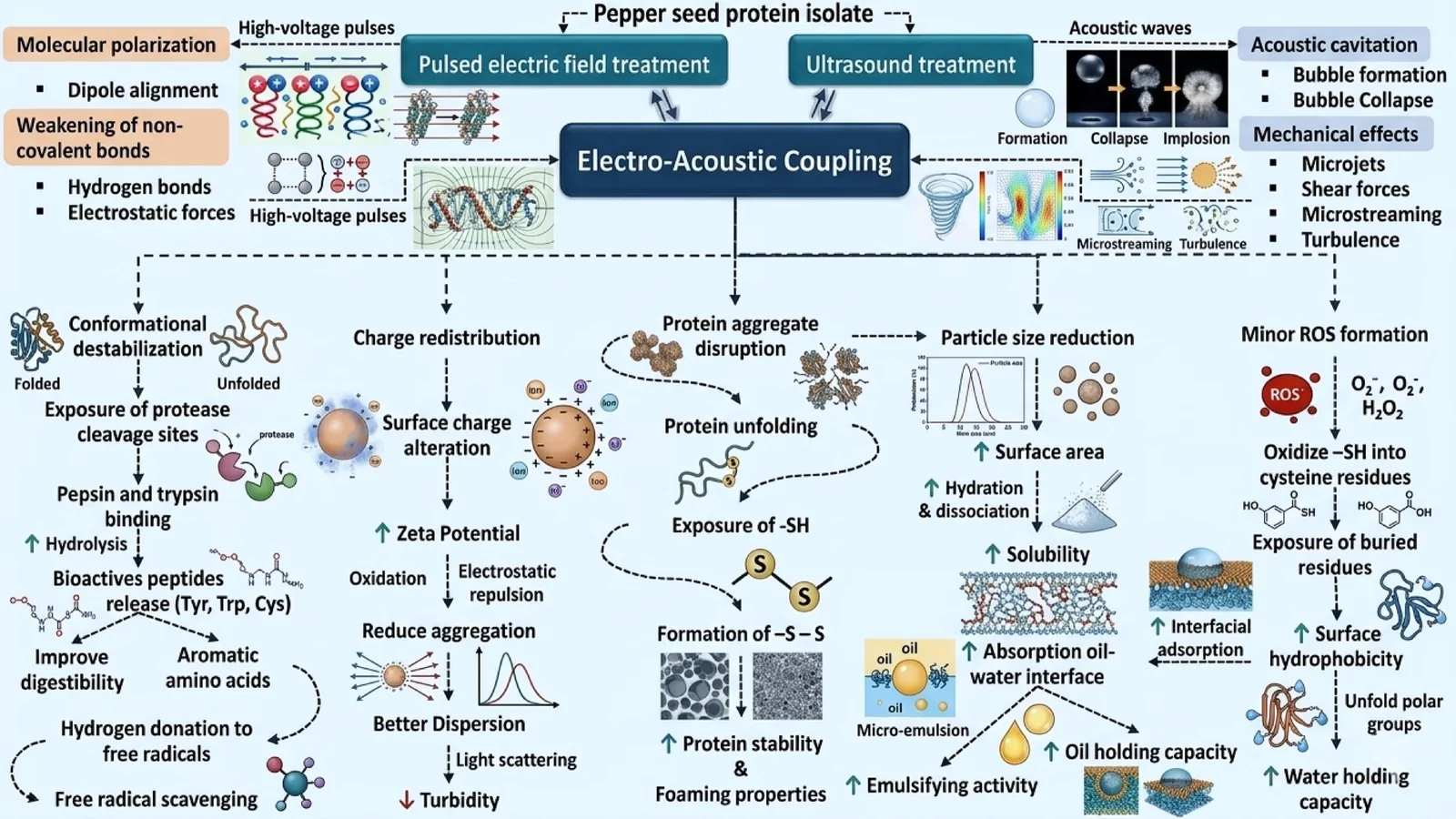
Mechanistic pathway of electro-acoustic coupling to modify the properties of PSPI.

The sequential US and PEF induce substantial structural and physicochemical modifications in PSPI, thereby enhancing digestibility, structural, functional, and antioxidant properties (Fig. 9). These modifications arise primarily from PEF voltage pulses, which induce potent electric fields in PSPI. These interact with polypeptides containing charged amino acids and dipole moments, resulting in dipole alignment, charge redistribution, and molecular polarization. This electrostatic disruption weakens the electrostatic forces, hydrogen bonds, and hydrophobic interactions in PSPI. Thus, PSPI undergoes conformational changes, with its tertiary structure becoming flexible but not yet completely unfolding, inducing protein aggregates that are more prone to other mechanical forces. Subsequent US processing induces acoustic waves that produce cavitation, described by the generation, growth, and ultimately collapse of microbubbles in PSPI. These bubble collapses induce localized pressure, intense shear forces, shockwaves, and microjets. These mechanical impacts break down the intermolecular interactions and aggregates, which PEF already destabilizes. US-induced cavitation disrupts aggregates, reduces particle size, and partially unfolds the proteins more efficiently. This integrated interaction, usually referred to as electro-acoustic coupling, improves the structural alteration beyond the impacts of individual PEF/US. Moreover, a small amount of ROS (∙OH and ∙O_2_^-^) is produced during cavitation as secondary products, which may contribute to mild oxidative changes in specific amino acid residues.

Electro-acoustic coupling disrupted aggregates and partially unfolded PSPI, exposing the previously buried functional groups, including sulfhydryl groups, hydrophobic residues, aromatic amino acids (Tyrosine, Trp) on the surface. These increase H_0_ and lead to rearrangement of the -S-S bond via binding between sulfhydryl groups. Modifications are also accompanied by secondary structural changes, including increases in random coil and α-helix content and a reduction in *β*-sheet content, indicating partial denaturation and molecular flexibility. The exposure of hydrophobic and hydrophilic groups enhances interactions with surrounding phases and protein hydration, thereby improving solubility. Moreover, it improves the rate of adsorption at oil-water interfaces, thereby increasing emulsifying ability. The increase in interfacial activity and flexibility leads to the formation of stable films around air bubbles, which increases foaming capacity. OHC and WHC may also increase due to the availability of nonpolar and polar binding sites.

Furthermore, partially unfolded protein improves *in vitro* digestibility by exposing protease cleavage sites that are buried in the native structure. This ultimately enhances accessibility, allowing trypsin and pepsin to hydrolyze polypeptide chains and facilitating the release of short peptides. The number of these peptides containing sulfur and aromatic amino acids with electron-donating properties, which neutralize ROS and scavenge free radicals, ultimately increased the antioxidant activity of PSPI.

However, the proposed electro-acoustic coupling mechanism should be interpreted as a plausible hypothesis rather than a directly validated phenomenon in the present study. Although the enhanced structural rearrangement and functional improvements observed after sequential PEF+US treatment suggest a synergistic interaction between the two non-thermal processes, the current experimental design does not allow direct confirmation of whether PEF pretreatment alters the subsequent cavitation behavior or acoustic energy transfer. Therefore, the proposed mechanism is primarily supported by the observed physicochemical changes and previous reports on the individual effects of PEF and US. Future investigations employing direct measurements of cavitation activity, acoustic pressure distribution, cavitation threshold, or sonochemical activity before and after PEF pretreatment are required to quantitatively validate the existence and contribution of electro-acoustic coupling during sequential PEF

+US processing.

## 4 Conclusion

The current study revealed that electro-acoustic coupling between sequential PEF+US treatment effectively tailors the structural, techno-functional, antioxidant properties, and *in vitro* digestibility of PSPI. The sequential treatment induced significant conformational changes, an increase in surface hydrophobicity, -SH groups, and zeta potential (-ve), along with a decrease in particle size. These modifications disrupted intermolecular aggregates and induced protein unfolding, thereby increasing electrostatic repulsion and ultimately improving colloidal stability. The reduction in fluorescence intensity with a distinct red shift indicated an increase in the aromatic residue’s exposure to a polar environment. FTIR analysis verified the rearrangement of secondary structure with increased random coil and α-helix contents, indicating improved molecular flexibility and protein unfolding. The scanning electron microscope confirmed these findings, showing significant fragmentation and disruption in SEM images. XRD results show a transition toward an amorphous structure and a reduction in crystallinity. DSC results showed a decrease in the enthalpy change, confirming reduced thermal stability due to the loss of ordered domains. Ultimately, the structural alterations are directly linked with better functional properties. The sequential treatment increased solubility and oil- and water-holding capacities, while decreasing turbidity. Foaming and emulsification properties improved due to increased molecular flexibility and interfacial adsorption. Increased antioxidant capacity was associated with greater exposure of reactive amino acid residues. Notably, *in vitro* digestibility increased significantly, which indicates increased enzymatic accessibility. The enhanced functional performance of PEF+US-treated PSPI demonstrates the potential of sequential PEF+US processing as a mild, non-thermal strategy for valorizing underutilized pepper seed protein into a sustainable, value-added food ingredient. Owing to its improved functional properties, the modified PSPI could be utilized in protein-fortified beverages, plant-based dairy and meat alternatives, emulsified products, and bakery formulations requiring enhanced protein functionality. From an energy-sustainability perspective, sequential PEF+US treatment may improve process efficiency by enhancing mass transfer and reducing processing time compared with individual PEF/US treatments. Previous literature has reported up to 37% lower overall energy consumption for PEF+US-assisted processing compared with untreated processing, highlighting its potential for more sustainable industrial-scale applications, although process-specific energy optimization is still required. Nevertheless, this study was conducted at a laboratory scale and primarily focused on structural and techno-functional characteristics. Therefore, future research should evaluate the bioavailability, sensory attributes, storage stability, and performance of modified PSPI in real food systems. Further investigations should also optimize treatment sequences, assess energy consumption and life-cycle impacts, and validate process scale-up. Moreover, the applicability of this sequential PEF+US strategy should be explored for other underutilized oilseed protein systems to broaden its industrial relevance and support sustainable plant protein utilization.

## Funding

This work was supported by the Deanship of Scientific Research, Vice Presidency for Graduate Studies and Scientific Research, King Faisal University, Saudi Arabia [GRANT: KFU261623].

## CRediT authorship contribution statement

**Muhammad Faisal Manzoor:** Writing – review & editing, Writing – original draft, Software, Methodology, Investigation, Formal analysis, Data curation, Conceptualization. **Xin-An Zeng:** Visualization, Supervision, Project administration, Funding acquisition. **Zahoor Ahmed:** Software, Data curation. **Rana Muhammad Aadil:** Software, Data curation. **Silvia del Carmen Beristain-Bauza:** Investigation. **Abderrahmane Aït-Kaddour:** Writing – original draft, Software, Data curation. **Muhammad Talha Afraz:** Software, Investigation, Data curation. **Marwa Ezz El-Din Ibrahim:** Software, Investigation, Data curation. **Muhammad Waseem:** Writing – original draft.

## Declaration of competing interest

The authors declare that they have no known competing financial interests or personal relationships that could have appeared to influence the work reported in this paper.
